# Comparative analysis of chloroplast genome structure and molecular dating in Myrtales

**DOI:** 10.1186/s12870-021-02985-9

**Published:** 2021-05-15

**Authors:** Xiao-Feng Zhang, Jacob B. Landis, Hong-Xin Wang, Zhi-Xin Zhu, Hua-Feng Wang

**Affiliations:** 1 Hainan Key Laboratory for Sustainable Utilization of Tropical Bioresources, College of Tropical Crops, Hainan University, Haikou, 570228 China; 2 School of Integrative Plant Science, Section of Plant Biology and the L.H. Bailey Hortorium, Cornell University, Ithaca, NY 14850 USA; 3 BTI Computational Biology Center, Boyce Thompson Institute, Ithaca, NY 14853 USA

**Keywords:** Myrtales, Plastome, Genome structure, Phylogeny, Adaptive evolution

## Abstract

**Background:**

Myrtales is a species rich branch of Rosidae, with many species having important economic, medicinal, and ornamental value. At present, although there are reports on the chloroplast structure of Myrtales, a comprehensive analysis of the chloroplast structure of Myrtales is lacking. Phylogenetic and divergence time estimates of Myrtales are mostly constructed by using chloroplast gene fragments, and the support for relationships is low. A more reliable method to reconstruct the species divergence time and phylogenetic relationships is by using whole chloroplast genomes. In this study, we comprehensively analyzed the structural characteristics of Myrtales chloroplasts, compared variation hotspots, and reconstructed the species differentiation time of Myrtales with four fossils and one secondary calibration point.

**Results:**

A total of 92 chloroplast sequences of Myrtales, representing six families, 16 subfamilies and 78 genera, were obtained including nine newly sequenced chloroplasts by whole genome sequencing. Structural analyses showed that the chloroplasts range in size between 152,214–171,315 bp and exhibit a typical four part structure. The IR region is between 23,901–36,747 bp, with the large single copy region spanning 83,691–91,249 bp and the small single copy region spanning 11,150–19,703 bp. In total, 123–133 genes are present in the chloroplasts including 77–81 protein coding genes, four rRNA genes and 30–31 tRNA genes.

The GC content was 36.9–38.9%, with the average GC content being 37%. The GC content in the LSC, SSC and IR regions was 34.7–37.3%, 30.6–36.8% and 39.7–43.5%, respectively. By analyzing nucleotide polymorphism of the chloroplast, we propose 21 hypervariable regions as potential DNA barcode regions for Myrtales. Phylogenetic analyses showed that Myrtales and its corresponding families are monophyletic, with Combretaceae and the clade of Onagraceae + Lythraceae (BS = 100%, PP = 1) being sister groups. The results of molecular dating showed that the crown of Myrtales was most likely to be 104.90 Ma (95% HPD = 87.88–114.18 Ma), and differentiated from the Geraniales around 111.59 Ma (95% HPD = 95.50–118.62 Ma).

**Conclusions:**

The chloroplast genome structure of Myrtales is similar to other angiosperms and has a typical four part structure. Due to the expansion and contraction of the IR region, the chloroplast genome sizes in this group are slightly different. The variation of noncoding regions of the chloroplast genome is larger than those of coding regions. Phylogenetic analysis showed that Combretaceae and Onagraceae + Lythraceae were well supported as sister groups. Molecular dating indicates that the Myrtales crown most likely originated during the Albian age of the Lower Cretaceous. These chloroplast genomes contribute to the study of genetic diversity and species evolution of Myrtales, while providing useful information for taxonomic and phylogenetic studies of Myrtales.

**Supplementary Information:**

The online version contains supplementary material available at 10.1186/s12870-021-02985-9.

## Background

**Fig. 1:**
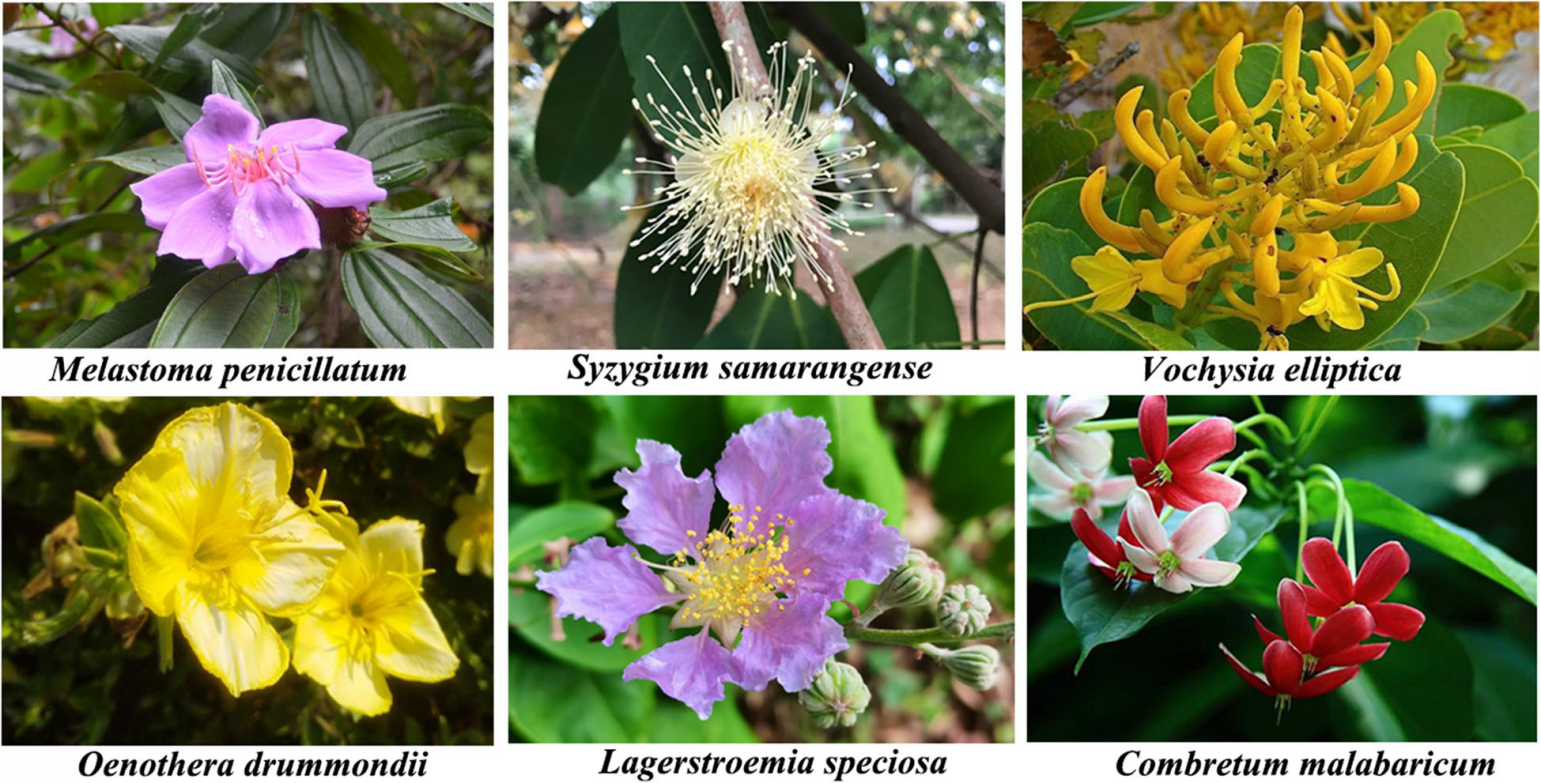
Flowers of typical plants in six families of Myrtales

The Myrtales belong to the Rosidae, which is one of the most speciose groups in the Rosanae clade of angiosperms [1, 2]. According to APG IV [3], Myrtales consists of nine families, 380 genera, and approximately 13,000 species. The nine families in the order are Alzateaceae, Combretaceae, Crypteroniaceae, Lythraceae, Melastomataceae, Myrtaceae, Onagraceae, Penaeaceae and Vochysiaceae. The species richness of families is unbalanced with relatively few species found in Alzateaceae, Crypteroniaceae and Penaeaceae. Species are widely distributed in the tropics, with Vochysiaceae showing an amphi-Atlantic disjunct distribution [2]. Species in Combretaceae are mainly distributed in tropical and subtropical regions, especially in African savannahs [4]. The order is morphologically diverse with herbaceous herbs, lianas, trees, and mangroves, as well as a wide variety of fruit types (berry, capsule, samara and drupe) [1] (Fig. 1). There are two main wood anatomical characteristics of Myrtales: bilateral vascular bundles in the primary stem and vascular bundles in the marginal depressions of secondary xylem, which are not common in other flowering plants. The combination of these two anatomical characteristics is exceedingly rare [5–7]. Many of the species of Myrtales have important economic [8], ornamental [9] and medicinal value [10, 11].

With the rapid development of second-generation sequencing technology, the cost of sequencing has made phylogenomic approaches feasible on large scales, ushering in a new exploration of plant identification and classification. Complete plastome sequences have become powerful tools to answer questions about plant evolution from inferred phylogenies [12–18]. The plastome is an essential organelle in photosynthetic cells, playing an important role in maintaining life [19] and is mainly maternally inherited in angiosperms. Most plastome DNA consist of double chains with a length of 120–220 kb [20] and a highly conserved typical four part genome structure. In recent years, researchers have been devoted to structural and phylogenetic analyses of chloroplasts in many groups, including Myrtales [21–23]. Structural characteristics of the chloroplasts have been useful for examining the genetic diversity and species evolution, and vital in developing policies for the protection of germplasm resources [24–26].

Reginato et al. [21] reported comparisons of chloroplast genomes in Melastomataceae for the first time. The structure, gene content and general characteristics of 16 chloroplast genomes of Melastomataceae and eight published chloroplast genomes of Myrtales were compared and analyzed. They found that the chloroplast genomes of Melastomataceae, like most angiosperms, have a typical tetrad structure with a large single copy region containing 84 protein coding genes (CDS), 37 tRNA and eight rRNA, for a total of 129 genes [21]. Gu et al. [22] reported the plastome of *Heimia myrtifolia*, an important medicinal plant with a variety of pharmacological alkaloids in the Lythraceae. Later, combined with 22 samples of other species in the Lythraceae, the chloroplast genome structure was comprehensively analyzed and compared with that of other species in Myrtales. The chloroplast genomes of 22 species of Lythraceae ranged from 152,049 bp to 160,769 bp, and included 10 variation hot spots that were selected as potential molecular markers [23]. In addition, other chloroplast genomes of Myrtales have been reported recently. Rodrigues et al. [27] compared the structure, gene number and genome size of six chloroplast genomes of Myrtales finding them to be similar to those of other Myrtales species. However, previous studies on chloroplast genomes of Myrtales have not been consistent, with some based on families, genera or species. Up to now, the comprehensive analysis of chloroplast genome structure of Myrtales is lacking.

In addition to studying the chloroplast genomes structure of Myrtales, researchers also explored the divergence time and phylogeny of Myrtales, but most studies were based on gene fragments. A strong phylogenetic framework is necessary to provide a basis for studying speciation. In previous molecular phylogenetic studies, a handful of chloroplast loci along with the internal transcribed spacer (ITS) and other ribosomal regions of nuclear DNA have been used for phylogenetic analysis of Myrtales [2, 7, 28]. Conti et al. [7] used 50 taxa (including 39 species and 11 outgroups) and the chloroplast gene *rbcL* to reconstruct the phylogeny of Myrtales. The results showed that Onagraceae and Lythraceae were closely related to Combretaceae [7]. Sytsma et al. [28] constructed the phylogenetic divergence time of Myrtales based on the chloroplast gene fragments *rbcL* and *ndhF* from 79 species of Myrtales and five fossil calibration points, indicating that Myrtales differentiated in the early Albian (111 Ma) with Combretaceae being the earliest branch of Myrtales with low support. Berger et al. [2] amplified and sequenced 6 gene fragments (*rbcL, ndhF, matK, matR,* 18S and 26S) from 102 taxa of Myrtales, and estimated the divergence time of Myrtales using 10 fossil calibration points. The results showed that the crown of Myrtales was most likely dated to 116 Ma (95% HPD = 113.7–118.8 Ma), while the phylogeny also showed that the Combretaceae is a sister group of all other families of Myrtales [2]. More recently, Li et al. [18] used 80 genes from 2881 plastomes and 62 fossil calibrations to reconstruct an angiosperm wide phylogeny showing that Myrtales and all of its families were monophyletic. The resulting phylogeny showed that the clade of Myrtales and Geraniales had a crown age of 112.26 Ma, as well as Combretaceae and Onagraceae + Lythraceae being sister groups with strong support. Most of the studies based on chloroplast gene fragments inferred relationships with low support, so using chloroplast genomes to explore the time of species differentiation and reconstruct phylogenetic relationship has credibility.

Currently there are few previous studies on the chloroplast genome structure of Myrtales. Although the phylogenetic position and relationships of Myrtales has been studied using molecular methods, the support for the placement of Myrtales is generally weak due to the lack of phylogenetic signal and sparse taxonomic sampling. Therefore, we set out to expand the sampling, reconstruct the phylogenetic relationship of Myrtales by using whole chloroplast genomes and comparatively analyze the plastome structure of Myrtales to provide the foundation for future research. In this study, we sequenced the chloroplast genomes of nine new species (including species of Myrtaceae, Melastomataceae and Combretaceae) and combined them with existing plastome data for Myrtales from NCBI to obtain a total of 95 chloroplast genomes, representing six families, 78 genera, and three outgroups. The main objectives of this study were to 1) analyze the chloroplast genome structure and elucidate the genetic diversity of Myrtales, 2) reconstruct the phylogenetic relationship of Myrtales to specifically determine the phylogenetic position of Combretaceae, and 3) infer the divergence time of Myrtales.

## Results

### Characteristics of chloroplast genomes

**Fig. 2:**
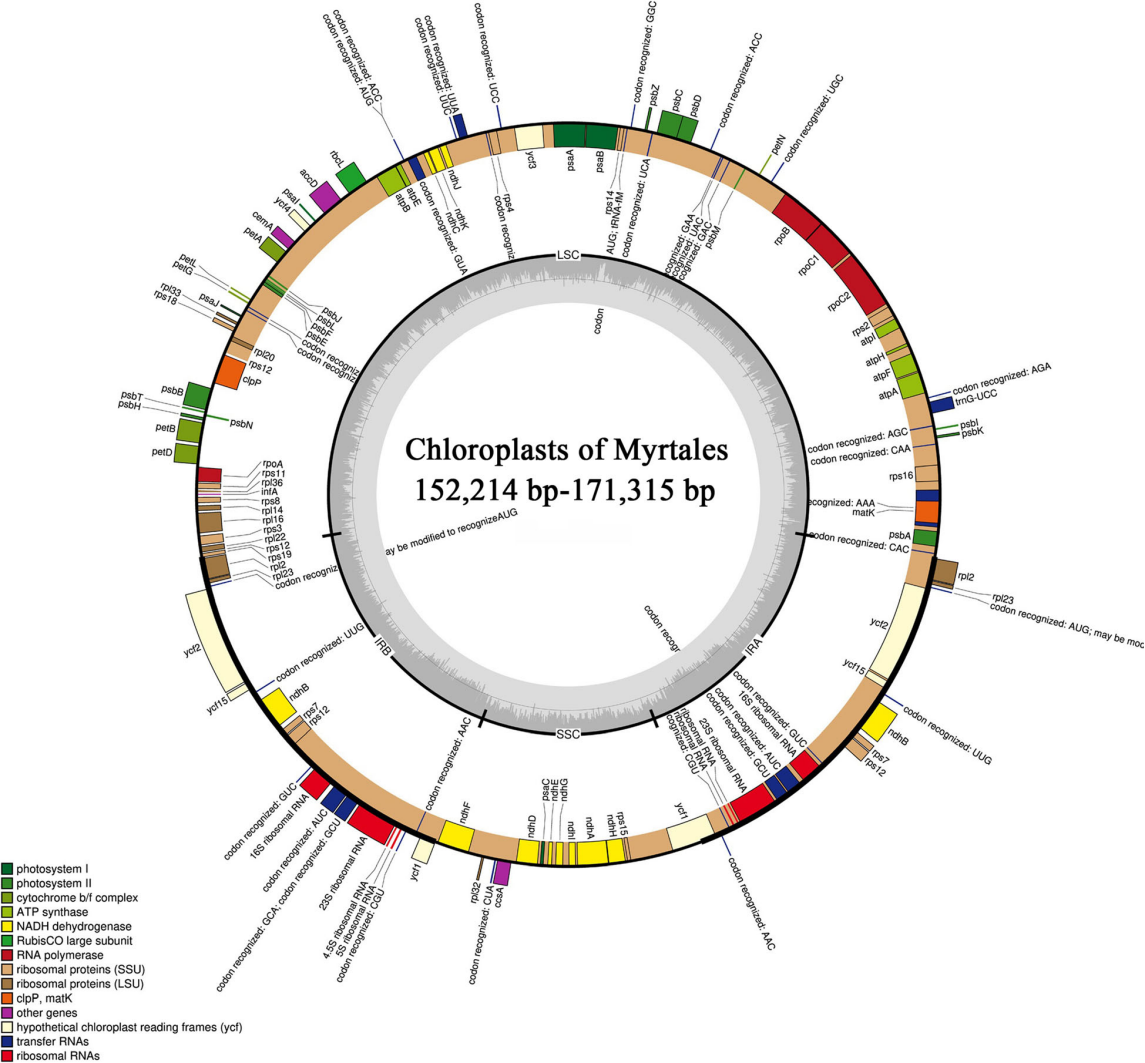
Chloroplast genome gene map of Myrtales. Genes on the inside of the outer circle are transcribed clockwise and those outsides are transcribed counterclockwise

Six families were represented with the 92 Myrtales chloroplast genomes used in this study: Melastomataceae (42 species in five subfamilies), Myrtaceae (including 19 species in five subfamilies), Vochysiaceae (seven species), Lythraceae (13 species in three subfamilies), Onagraceae (three species in two subfamilies), and Combretaceae (eight species in one subfamily). All chloroplast genomes have a typical four part structure: large single copy region (LSC), small single copy region (SSC) and two inverted repeat regions (IRs) (Fig. 2). The length of the chloroplast genomes in the 42 samples of Melastomataceae ranged from 153,304 bp (*Sarcopyramis napalensis*, MK994868.1) to 157,991 bp (*Astronia smilacifolia*, MK994883.1), while the 19 samples of Myrtaceae ranged from 156,129 bp (*Rhodomyrtus tomentosa*, NC_043848.1) to 160,459 bp (*Eucalyptus grandis*). The chloroplast genomes of the seven Vochysiaceae samples ranged in length from 160,687 bp (*Erisma bracteosum*, NC_043794.1) to 171,315 bp (*Vochysia acuminata*, NC_043811.1), the 13 Lythraceae samples ranged from 152,214 bp (*Lagerstroemia excelsa*, NC_042896.1) to 160,054 bp (*Pemphis acidula*, NC_041439.1), and the three Onagraceae samples ranged from 159,396 bp (*Ludwigia octovalvis*, NC_031385.1) to 165,779 bp (*Oenothera villaricae*, NC_030532.1). Finally, the length of the chloroplast genomes in the eight samples of Combretaceae ranged from 159,750 bp (*Terminalia guyanensis*, NC_043807.1) to 161,773 bp (*Combretum littoreum*). Across all chloroplast genomes of Myrtales, the difference in plastome size between families was 19,101 bp, the difference of the IR region was 12,846 bp, the difference of the SSC region was 8553 bp, and the difference of the LSC region was 7558 bp. All 92 chloroplast genomes showed a typical quadripartite structure, comprising two IR regions (26,781–36,747 bp) separated by the LSC (83,691–91,249 bp) and the SSC (11,150–19,703 bp) regions (Table 1). In addition, a total of 123–133 genes are encoded, of which 106–116 are single copy with 17 genes duplicated in the IR regions. Of the unique genes 77–81 are protein coding genes, 29–31 are tRNA genes, and four are rRNA genes. The total GC content of the chloroplast genomes are highly similar (36.9–38.9%), with the average GC content across the entire chloroplast genomes being 37%, while the different regions had slightly variable GC content with the LSC, SSC and IR ranging from 34.7–37.3%, 30.6–36.8%, and 39.7–43.5%, respectively (Tables 1 and 2).

**Table 1:** Summary of major characteristics of plastomes in Myrtales and related outgroups

Species name	lastomes GenBank accession number	Genome size (bp)	LSC Length (bp)	SSC Length (bp)	IR Length (bp)	Number of genes			G+C(%)			
						CDS	tRNAs	rRNAs	Total genome	LSC	SSC	IR
*Allomaieta villosa*	NC_031875.1	156452	85915	16975	26781	80	30	4	36.90%	34.70%	30.60%	42.50%
*Scorpiothyrsus shangszeensis*	MK994866.1	156371	85899	16902	26785	80	30	4	36.90%	34.70%	30.60%	42.50%
*Sonerila borneensis*	MK994893.1	154804	84872	16480	26726	80	30	4	37.30%	35.10%	31.00%	42.60%
*Sporoxeia petelotii*	MK994904.1	156529	86026	17037	26733	80	30	4	36.90%	34.80%	30.50%	42.50%
*Styrophyton caudatum*	MK994860.1	156386	85920	16930	26768	80	30	4	36.90%	34.70%	30.40%	42.50%
*Tibouchina longifolia*	NC_031889.1	156789	86297	17124	26684	81	30	4	37.10%	34.90%	31.10%	42.50%
*Tigridiopalma magnifica*	NC_036021.1	155663	85161	16932	26785	79	31	4	37.10%	35.00%	30.70%	42.50%
*Triolena amazonica*	NC_031890.1	156652	86200	16970	26741	80	30	4	36.90%	34.70%	30.70%	42.50%
*Anerincleistus bracteatus*	MK994899.1	156862	86293	16989	26790	79	30	4	37.00%	34.80%	30.60%	42.50%
*Barthea barthei*	MK994907.1	155948	85540	16808	26791	79	30	4	37.00%	34.80%	30.50%	42.50%
*Bertolonia acuminata*	NC_031876.1	156045	85571	17008	26733	80	30	4	37.00%	34.70%	30.80%	42.50%
*Blakea schlimii*	NC_031877.1	155862	85370	16998	36747	80	30	4	37.10%	34.90%	30.90%	42.50%
*Blastus cochinchinensis*	MK994909.1	155969	85900	16445	26812	79	30	4	37.00%	34.80%	30.70%	42.40%
*Bredia okinawensis*	MK994873.1	156023	85502	16925	26798	79	30	4	37.00%	34.80%	30.50%	42.50%
*Cyphotheca montana*	MK994852.1	156422	85898	16972	26776	79	30	4	37.00%	34.80%	30.60%	42.50%
*Dissochaeta beccariana*	MK994889.1	156285	85955	16933	26702	79	30	4	36.90%	34.60%	30.80%	42.50%
*Driessenia phasmolacuna*	MK994923.1	156620	86031	17055	26767	79	30	4	36.80%	34.60%	30.30%	42.50%
*Fordiophyton jinpingense*	MK994875.1	154430	84239	16799	26696	79	30	4	37.20%	35.10%	30.70%	42.50%
*Macrolenes pachygyna*	MK994894.1	156366	85966	16893	26754	79	30	4	37.00%	34.80%	30.80%	42.50%
*Medinilla speciosa*	MK994885.1	155084	84768	16752	26782	79	30	4	37.00%	34.80%	30.70%	42.50%
*Melastoma candidum*	NC_034716.1	156682	86084	17094	26752	79	29	4	37.20%	35.00%	31.20%	42.50%
*Merianthera pulchra*	NC_031881.1	156168	85621	17001	26773	80	30	4	37.00%	34.80%	30.70%	42.40%
*Microlicia cogniauxiana*	NC_043792.1	155732	90463	19043	23902	79	30	4	37.00%	34.90%	33.30%	43.30%
*Nepsera aquatica*	NC_031883.1	155110	84644	17066	26700	80	30	4	37.10%	34.80%	31.00%	42.60%
*Sarcopyramis napalensis*	MK994868.1	153304	83691	16153	26730	79	30	4	37.00%	34.80%	30.50%	42.50%
*Ochthocharis bornensis*	MK994895.1	156672	86033	17101	26769	79	30	4	36.90%	34.70%	30.70%	42.50%
*Opisthocentra clidemioides*	NC_031884.1	156352	85866	16942	26772	80	30	4	37.00%	34.80%	30.90%	42.50%
*Oxyspora teretipetiolata*	MK994853.1	156303	85767	17000	26768	79	30	4	36.90%	34.70%	30.50%	42.50%
*Phyllagathis suberalata*	MK994928.1	156075	85429	17114	26766	79	30	4	37.00%	34.80%	30.50%	42.50%
*Plagiopetalum serratum*	MK994902.1	156181	85924	16783	26737	79	30	4	37.00%	34.80%	30.70%	42.50%
*Pterogastra divaricata*	NC_031885.1	154948	84718	17156	26537	79	30	4	37.20%	35.10%	31.20%	42.50%
*Rhexia virginica*	NC_031886.1	154635	84459	16924	26626	80	30	4	37.20%	35.10%	31.10%	42.50%
*Rhynchanthera bracteata*	NC_031887.1	155108	85093	16729	26643	80	30	4	37.00%	34.70%	30.70%	42.60%
*Tibouchina semidecandra*	HCNGB, RL0146	155544	85204	17252	26544	79	30	4	37.00%	34.90%	31.10%	42.40%
*Salpinga maranonensis*	NC_031888.1	153311	85128	16653	25765	79	29	4	37.40%	35.30%	31.70%	42.80%
*Miconia dodecandra*	NC_031882.1	157216	86609	16999	26804	80	30	4	37.00%	34.80%	31.00%	42.50%
*Eriocnema fulva*	NC_031878.1	155994	85431	16953	26805	80	30	4	37.00%	34.80%	30.80%	42.50%
*Graffenrieda moritziana*	NC_031879.1	155733	85341	16924	26734	79	30	4	37.00%	34.70%	30.90%	42.50%
*Henriettea barkeri*	NC_031880.1	156527	85991	17036	26750	80	30	4	36.90%	34.70%	30.60%	42.50%
*Astronia smilacifolia*	MK994883.1	157991	87376	17074	26765	79	30	4	36.90%	34.70%	30.80%	42.50%
*Memecylon ligustrifolium*	MK994913.1	157154	86723	17026	26735	79	30	4	37.10%	34.90%	31.00%	42.50%
*Pternandra korthalsiana*	MK994877.1	157496	86730	17358	26747	79	30	4	37.00%	34.90%	30.90%	42.30%
*Rhodomyrtus tomentosa*	NC_043848.1	156129	86298	18183	25824	79	30	4	38.10%	35.10%	30.80%	42.90%
*Psidium guajava*	NC_033355.1	158841	87675	18464	26351	79	30	4	37.00%	34.90%	30.70%	42.80%
*Plinia cauliflora*	NC_039395.1	159095	88182	18615	26159	79	30	4	37.00%	34.80%	30.80%	42.70%
*Campomanesia xanthocarpa*	KY392760.1	158131	87596	18595	25970	78	30	4	37.00%	34.80%	30.60%	42.90%
*Acca sellowiana*	KX289887.1	159370	88028	18598	26372	80	30	4	37.00%	34.90%	30.60%	42.80%
*Stockwellia quadrifida*	NC_022414.1	159561	88247	18544	26385	79	30	4	36.90%	34.70%	30.70%	42.70%
*Eucalyptus grandis*	HCNGB, RL0106	160459	88939	18750	26385	81	30	4	36.80%	34.70%	36.80%	42.70%
*Eucalyptus microcorys*	NC_022404.1	160225	89051	18410	26382	79	30	4	36.80%	34.70%	30.50%	42.70%
*Eucalyptus erythrocorys*	NC_022406.1	159742	88691	18287	26382	79	30	4	36.90%	34.70%	30.40%	42.70%
*Corymbia tessellaris*	NC_022410.1	160127	88617	18692	26409	79	30	4	36.80%	34.60%	30.50%	42.70%
*Corymbia maculata*	NC_022408.1	160045	88557	18670	26409	79	30	4	36.80%	34.60%	30.50%	42.70%
*Corymbia eximia*	NC_022409.1	160012	88522	18672	26409	79	30	4	36.80%	34.60%	30.50%	42.70%
*Angophora floribunda*	NC_022411.1	160245	88715	18746	26392	79	30	4	36.80%	34.50%	30.50%	42.70%
*Angophora costata*	NC_022412.1	160326	88769	18773	26392	79	30	4	36.80%	34.50%	30.50%	42.70%
*Allosyncarpia ternata*	NC_022413.1	159593	88218	18571	26402	79	30	4	37.50%	34.60%	30.50%	42.70%
*Heteropyxis natalensis*	NC_043799.1	159859	87884	18919	26528	79	30	4	36.90%	34.80%	30.70%	42.70%
*Syzygium forrestii*	HCNGB, RL0700	159996	88560	18608	26414	80	30	4	36.90%	34.80%	30.80%	42.60%
*Syzygium cumini*	HCNGB, RL0850	159996	88560	18608	26414	79	30	4	36.90%	34.80%	30.80%	42.60%
*Melaleuca leucadendra*	HCNGB, RL0233	160317	88776	18619	26461	80	30	4	36.70%	34.50%	30.40%	42.50%
*Ruizterania albiflora*	NC_043804.1	162345	90200	19417	28364	79	30	4	36.50%	34.20%	30.30%	42.70%
*Vochysia acuminata*	NC_043811.1	171315	91249	11150	34457	79	30	4	35.90%	33.80%	30.60%	39.70%
*Salvertia convallariodora*	NC_043806.1	171267	91243	11152	34435	79	30	4	35.90%	33.80%	30.60%	39.70%
*Qualea grandiflora*	NC_043803.1	161026	90880	18260	26443	79	30	4	36.50%	34.20%	30.40%	42.70%
*Callisthene erythroclada*	NC_043793.1	161626	89825	19351	26225	79	30	4	36.70%	34.50%	30.50%	42.70%
*Korupodendron songweanum*	NC_043798.1	161149	88587	18640	26956	78	30	4	36.60%	34.40%	30.40%	42.40%
*Erisma bracteosum*	NC_043794.1	160687	89210	18740	26369	79	30	4	36.40%	34.20%	30.30%	42.40%
*Duabanga grandiflora*	NC_042899.1	156084	86467	16502	26556	80	30	4	37.50%	35.60%	31.30%	42.50%
*Lagerstroemia calyculata*	NC_042897.1	152294	84012	16798	25742	80	30	4	37.70%	36.00%	31.20%	42.50%
*Lagerstroemia excelsa*	NC_042896.1	152214	84053	16917	25622	80	30	4	37.60%	35.90%	31.00%	42.50%
*Lagerstroemia venusta*	NC_042892.1	152521	84194	16833	25747	80	30	4	37.60%	35.90%	31.00%	42.50%
*Lawsonia inermis*	NC_042369.1	157755	88423	17386	25973	80	30	4	36.90%	34.80%	31.00%	42.50%
*Sonneratia alba*	NC_039975.1	153061	87226	18033	23901	80	29	4	37.30%	35.40%	31.10%	43.10%
*Trapa maximowiczii*	NC_037023.1	155577	88528	18273	24388	78	31	4	36.40%	34.20%	30.20%	42.80%
*Trapa natans*	NC_042895.1	155553	88472	18274	24387	80	30	4	36.40%	34.20%	30.20%	42.80%
*Lythrum salicaria*	NC_042891.1	158483	88997	18530	25477	80	30	4	36.80%	34.80%	30.70%	42.60%
*Heimia apetala*	NC_043797.1	159218	88570	18822	25913	79	30	4	37.00%	35.00%	30.60%	42.60%
*Pemphis acidula*	NC_041439.1	160054	89785	18883	25693	80	30	4	36.50%	34.30%	29.70%	42.70%
*Punica granatum*	NC_035240.1	158633	89017	18686	25465	79	30	4	36.90%	34.90%	30.60%	42.80%
*Woodfordia fruticosa*	NC_042898.1	159380	89569	18697	25557	80	30	4	36.60%	34.50%	30.20%	42.70%
*Oenothera villaricae*	NC_030532.1	165779	87891	16200	30844	78	31	4	38.90%	37.30%	35.30%	42.10%
*Epilobium ulleungensis*	NC_039575.1	160912	88915	17327	27335	80	30	4	38.20%	36.30%	33.20%	42.80%
*Ludwigia octovalvis*	NC_031385.1	159396	90183	19703	24755	77	30	4	37.40%	35.20%	32.00%	43.50%
*Terminalia guyanensis*	NC_043807.1	159750	88671	18413	26333	79	30	4	37.00%	34.70%	30.80%	43.00%
*Lumnitzera racemosa*	NC_042408.1	159473	88056	18613	26402	79	30	4	37.00%	34.70%	30.70%	42.90%
*Lumnitzera littorea*	NC_039752.1	159687	88323	18558	26403	79	30	4	37.00%	34.70%	30.90%	43.00%
*Laguncularia racemosa*	NC_042719.1	158311	87022	18886	26247	79	30	4	37.00%	34.80%	30.30%	43.00%
*Combretum kraussii*	HCNGB, RL0855	154081	85457	17093	25734	81	30	4	37.40%	35.50%	31.00%	42.80%
*Combretum littoreum*	HCNGB, RL0942	161773	90179	18730	26432	79	30	4	37.10%	34.80%	30.90%	43.00%
*Terminalia catappa*	B244	159,873	88,794	18,013	26,533	80	30	4	36.90%	30.90%	30.90%	42.80%
*Combretum malabaricum*	B246	159,425	88,399	17,848	26,589	80	30	4	37.20%	35.00%	31.10%	42.90%
*Viviania marifolia*	NC_023259.1	157291	83138	4551	34801	72	30	4	37.70%	35.80%	29.20%	40.40%
*Pelargonium tetragonum*	NC_031205.1	173410	75181	6764	45736	82	30	4	39.80%	38.40%	34.70%	41.40%
*Pelargonium quercifolium*	NC_031203.1	170569	87543	6706	38163	78	30	4	39.00%	38.00%	33.80%	40.60%

**Table 2:** Average length and G + C content for complete chloroplast genomes of the subfamilies in Myrtales

family	Number of species	Average length (bp)				Average G + C content (%)			
		Genome	LSC	SSC	IR	LSC	SSC	IR	Genome
Melastomataceae	42	159,995	85,754	16,984	26,888	34.86	30.84	42.52	37.02
Myrtaceae	19	159,583	88,310	18,596	26,339	34.72	30.93	42.71	36.97
Vochysiaceae	7	164,202	90,171	16,673	29,036	34.16	30.44	41.76	36.36
Lythraceae	13	156,217	87,486	17,895	25,417	34.5	30.2	42.7	36.6
Onagraceae	3	162,030	88,996	17,743	27,645	36.27	33.5	42.8	38.17
Combretaceae	8	159,047	88,113	18,269	26,334	37.08	34.39	30.83	42.93

### Boundaries between IR and SC regions

**Fig. 3:**
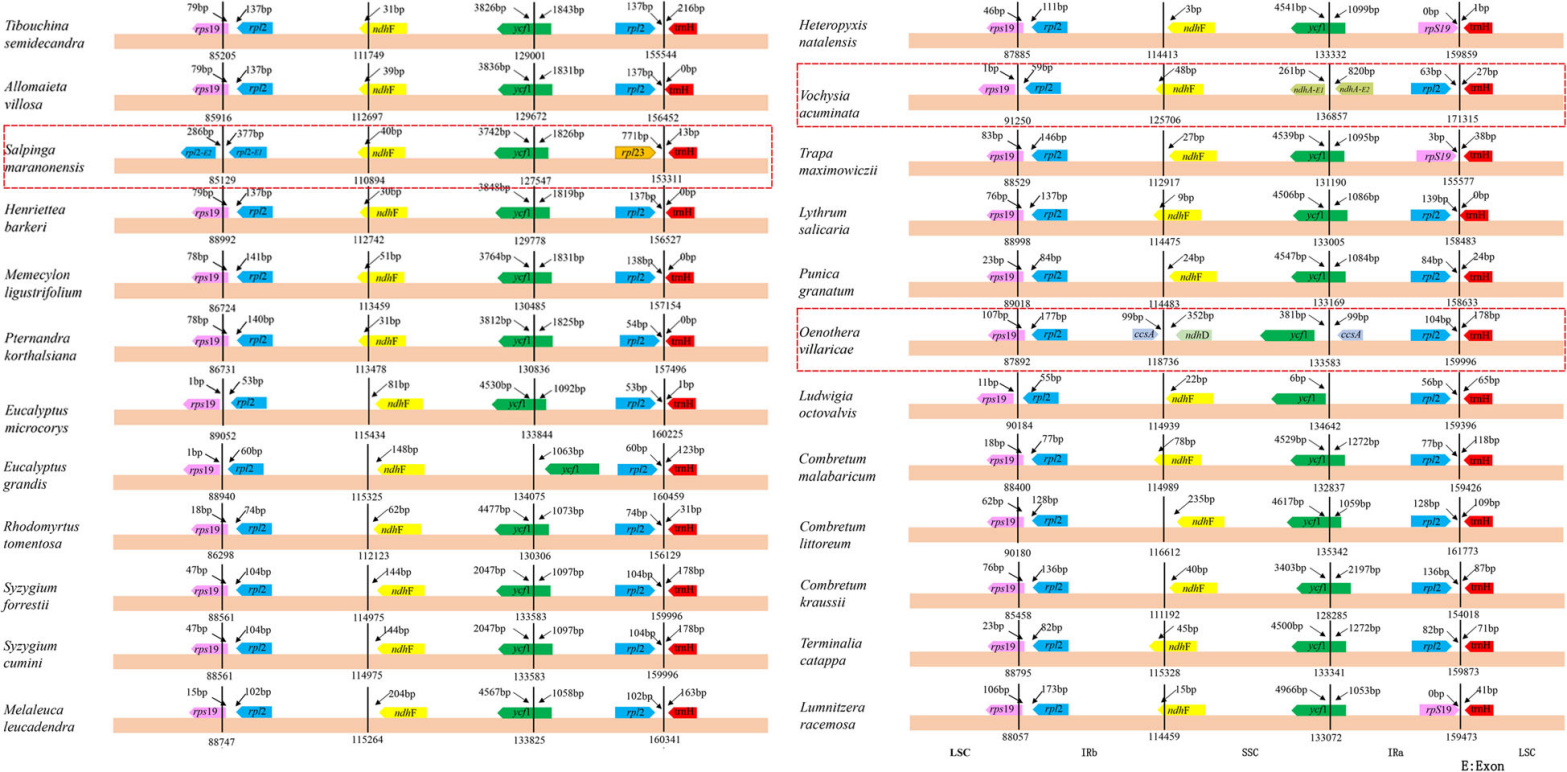
Comparison of the IR/SC junctions among 24 chloroplast genomes of Myrtales (15 samples from NCBI and the nine newly sequenced chloroplast genomes covering 16 subfamilies/families within Myrtales)

In total, we analyzed and compared the differences between boundary regions of the SC and IR in 24 chloroplast genomes (15 samples from NCBI and the nine newly sequenced chloroplast genomes covering 16 subfamilies/families within Myrtales). We found that most chloroplast genomes have similar characteristics. The junction of the LSC/IRb region of 23 chloroplast genomes was located at the *rps19* and *rpl2* genes, while the junction of LSC/IRb region of *Salpinga maranonensis* (NC_031888.1) was unique with the boundary at the *rpl2* gene. Except for *Oenothera villaricae* (NC_ 030532.1) the boundary of IRb/SSC was *ccsA* - *ndhD.* The *ndhF* gene was detected at the boundary of IRb/SSC in all other species. The *ndhF* gene of 11 species crossed the boundary of IRb/SSC, while *ndhF* of 12 species was completely found in the SSC region, ranging between 3 and 235 bp from the boundary. The gene *ycf1* is at the SSC/IRa boundary except in *Vochysia acuminata* (NC_043811.1) and *Oenothera villaricae* (NC_030532.1). In total there are 20 species for which *ycf1* crosses the boundary between SSC/IRa, two species in which *ycf1* is completely in the SSC ranging from 63 to 381 bp away from the boundary, and one species in which *ycf1* is completely in the IRa 1063 bp away from the boundary. The genes *rpl2* and *trnH* (*rpl2* is located in IRa, 53–139 bp away from the boundary, *trnH* is located in LSC, 0–216 bp away from the boundary) were detected in the IRa/LSC boundary for 20 species. The genes *rps19* and *trnH* (*rps19* is located in IRa, 0–3 bp away from the boundary, *trnH* is located in LSC, 1–41 bp away from the boundary) were detected in the IRa/LSC boundary for three species, and *rpl23* and *trnH* were detected in the IRa/LSC boundary for *Salpinga maranensis* (NC_031888.1) (Fig. 3).

### Comparative genomic analysis and divergence hotspot regions

**Fig. 4:**
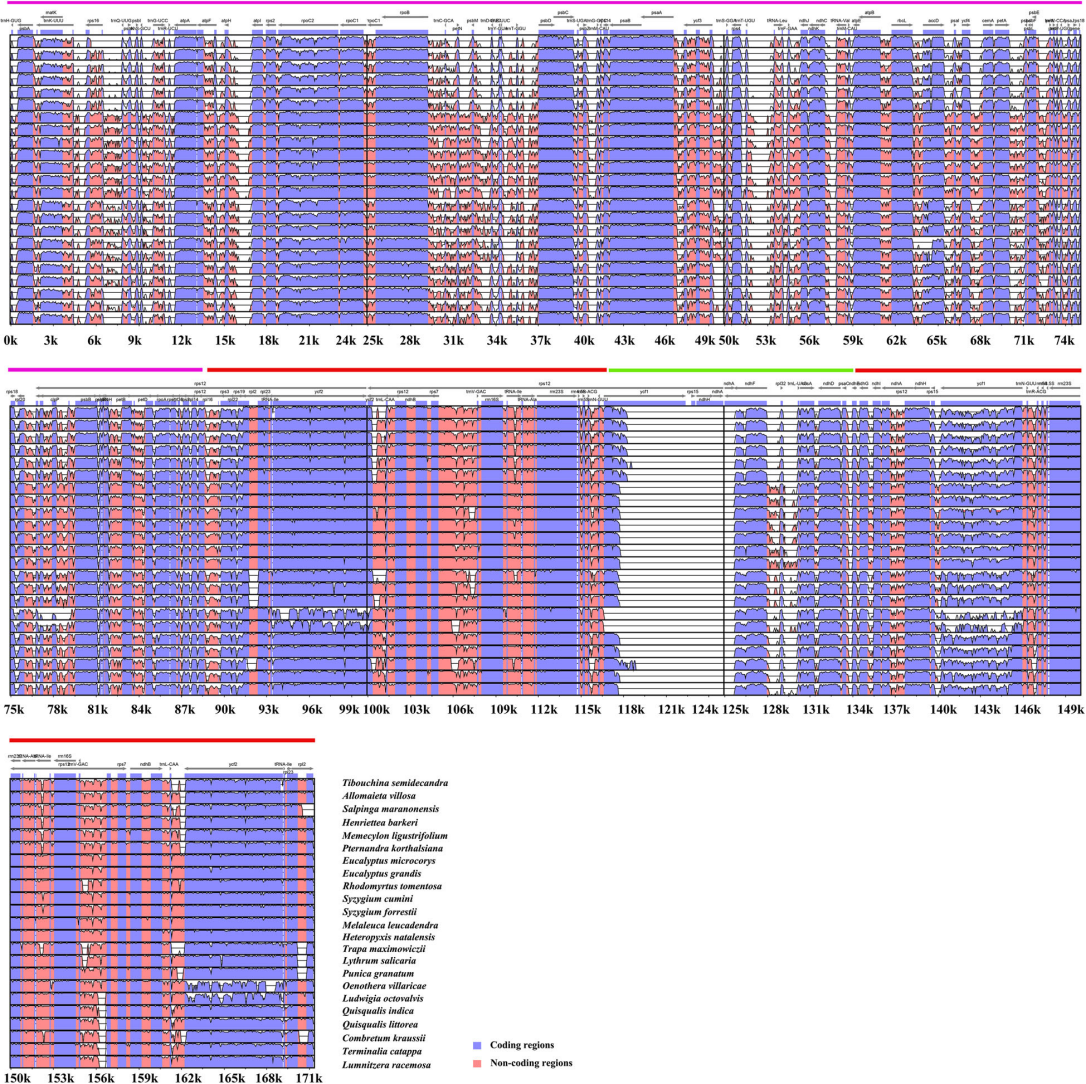
Visualization of the alignment of 24 chloroplast genome sequences of Myrtales. The plastome of *Vochysia acuminata* was used as the reference. The Y-axis depicts percent identity to the reference genome (50–100%) and the X-axis depicts sequence coordinates within the plastome. Genome regions were color-coded according to coding and non-coding regions

**Fig. 5:**
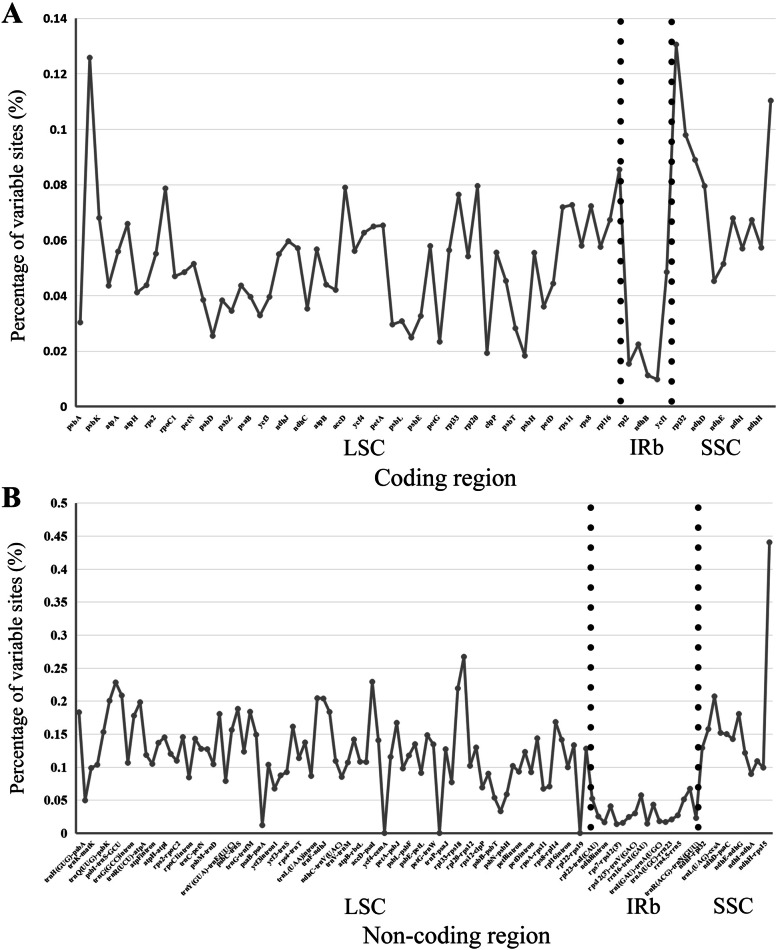
Comparison of the nucleotide diversity values across 92 chloroplast genomes of Myrtales. **a** Protein-coding regions. **b** Noncoding regions. The vertical dotted lines divides the approximate boundary of LSC, IRb and SSC

We analyzed the comprehensive sequence divergence of the 24 Myrtales chloroplast genomes (15 samples from NCBI and the nine newly sequenced chloroplast genomes covering 16 subfamilies/families within Myrtales) using the mVISTA software with the annotation of *V. acuminate* used as a reference. A genome wide alignment revealed globally high sequence similarity (> 90% identity) (Fig. 4). The LSC and SSC regions show a higher level of sequence divergence than the inverted repeat regions. In addition, 188 regions were extracted to calculate nucleotide variability (Table S1). In coding regions, the loci with the largest variation are *matK, rpoC2, accD, rpl20, ndhF, rpl32, ccsA*, *ndhD*, and *rps15*; in non-coding regions, the loci with the largest variation are *psbK-psbI, psbI-trnS (GCU), trnS (GCU)-trnG (GCC), trnR (UCU)-atpA, psbC-trnS (GCU), trnG-trnfM, trnF-ndhJ, ndhJ-ndhK, accD-psaI, rpl33-rps18, rps18-rpl20* and *rps15-ycf1*. DNA barcodes with the largest nucleotide diversity are considered to be the focus of phylogenetic analysis and plant identification (Fig. 5).

### Phylogenetic results

**Fig. 6:**
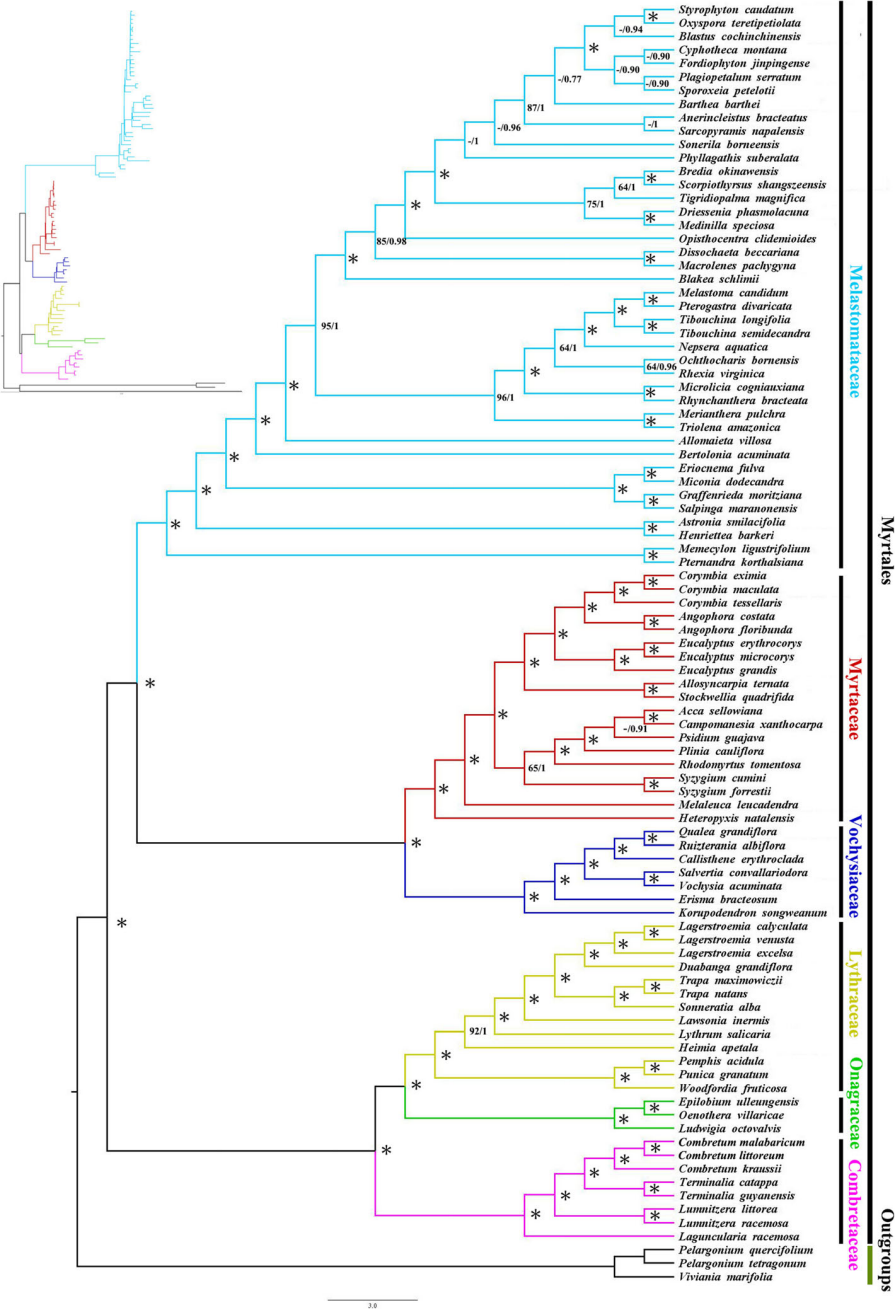
Optimal phylogenetic tree resulting from analyses of 92 complete chloroplast genomes of Myrtales and 3 outgroups using Maximum Likelihood (ML) and Bayesian inference (BI). Support values are maximum likelihood bootstrap support/Bayesian posterior probability; asterisks indicate 100%/1.0 support values. The families of Myrtales are indicated by different colors. The inset shows the same tree as a phylogram

Both ML and BI analyses of the complete chloroplast generated almost identical topologies with strong support at every node [ML bootstrap (BS) = 100%, Bayesian posterior probabilities (PP) = 1] (Fig. 6). Melastomataceae, Myrtaceae, Vochysiaceae, Onagraceae, Lythraceae, and Combretaceae were fully supported as monophyletic, with Combretaceae resolved as sister to Onagraceae + Lythraceae clade (BS/PP = 100/1; (Fig. 6). Melastomataceae was recovered as sister to Myrtaceae + Vochysiaceae (BS/PP = 100/1). A clade of Melastomataceae + Myrtaceae + Vochysiaceae was recovered as sister to the clade of Combretaceae + Onagraceae + Lythraceae with strong support (BS/PP = 100/1). In addition, the phylogenetic trees constructed using the coding regions (CR), noncoding regions (NCR), LSC, SSC and NO-IRa phylogenetic trees (ML / BI) have the same topological structure at the family level as the phylogeny inferred from the full chloroplast with strong support (Figure S1, S2, S3. S4 and S5). Observed differences were found in the phylogenetic relationships constructed by the IRb region, in which Melastomataceae was resolved as sister to Myrtaceae + Vochysiaceae + Lythraceae + Combretaceae, and Lythraceae was resolved as a sister to Combretaceae albeit with low support (Figure S6). Additionally, we expanded the outgroups to construct the phylogenetic relationship of Malvids, and the phylogenetic relationship of Myrtales was also strongly supported (Figure S7).

### Divergence time estimation of Myrtales

**Fig. 7:**
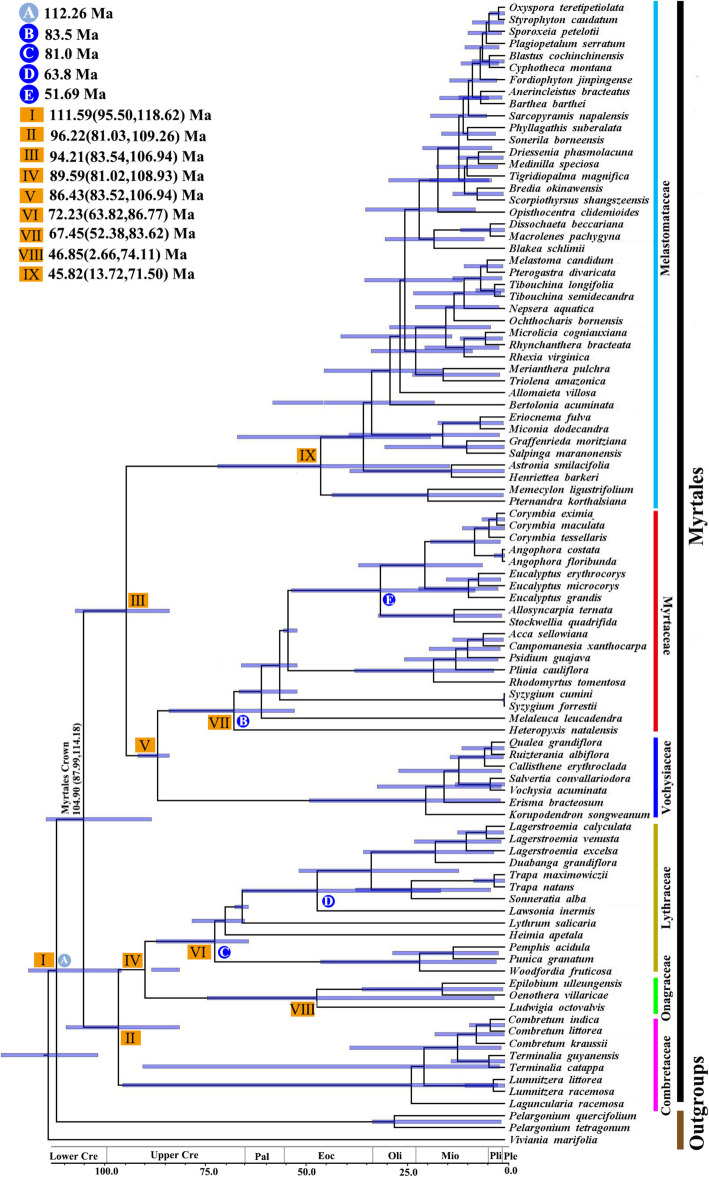
Chronogram of Myrtales based on complete chloroplast genomes sequences estimated from BEAST. The blue circle represents four fossil constraints and one grey circle represents secondary constraint, and the yellow boxes represent our estimated divergence times of major lineages

The results of the BEAST analysis of species divergence time in Myrtales are shown in Fig. 7. The crown age of Myrtales is 104.90 Ma (95% HPD = 87.88–114.18 Ma) with the recent common ancestor with Geraniales dated to 111.59 Ma (95% HPD = 95.50–118.62 Ma) during the Albian age of the Lower Cretaceous. Based on the BEAST chronogram, the Combretaceae with Onagraceae + Lythraceae (crown group age: 89.59 Ma, HPD = 81.02-108.93 Ma) diverged 96.22 Ma (95% HPD = 81.03–109.26 Ma) in the Cenomanian age of the Upper Cretaceous. The crown group of Melastomataceae (crown group age: 45.82 Ma, 95% HPD = 13.72–71.50 Ma) with Myrtaceae + Vochysiaceae (crown group age: 86.43 Ma, 95% HPD = 83.52–106.94 Ma) diverged at 94.21 Ma (95% HPD = 83.54–106.94 Ma) in the Cenomanian age of the Upper Cretaceous.

## Discussion

### Plastome structure comparisons and sequence divergence hotspots

Previous studies have shown that the size of chloroplast genomes in angiosperms are between 120 and 180 kb, and the size of IR region is 20–30 kb [29]. The size range of the 92 chloroplast genomes in Myrtales is 152,214–171,315 bp, of which the IR is 26,781–36,747 bp. Our results show that the chloroplast genomes of Myrtales are on the larger end of organellar genomes in angiosperms. The largest plastome is in the Vochysiaceae, and the smallest plastome is in the Lythraceae. The difference of plastome length between different families mainly lies in the difference of IR region length. The change in the overall length of chloroplast genomes is generally related to the expansion and contraction of IR regions [30]. The presented results are similar to those found in *Pelargonium hortorum*, *Cryptomeria fortunei*, *Geranium*, *Pisum sativum*, *Vicia faba*, and *Erodium* in which the size of the IR is increased, decreased or even completely lost [31–34]. In angiosperms, high conservation of the IR region is common, and is important for stabilizing plastome gene structure [35] though changes have been reported including in some early diverging eudicots [36, 37].

The nucleotide content of chloroplasts is relatively stable and the gene structure is highly conserved, though mutation hotspots do exist. Genes with a relatively high mutation rate can be used as DNA barcodes to help distinguish between accessions within a given taxon [38, 39] and varieties in germplasm resources [40, 41]. In this study, we used mVISTA to compare the whole chloroplast of 24 species of Myrtales and used DnaSP to analyze the percentage of variable loci in 74 coding genes and 114 non-coding regions. Similar to previous results, the variation of noncoding regions is greater than that of coding regions [42, 43]. As observed in members of Adoxaceae and *Panax notoginseng*, the variation of the IR region of Myrtales is smaller than that of the SC region [44, 45]. Previous studies investigating the phylogeny of Myrtales using only *rbcL* failed to resolve the phylogenetic position of the order. Our analyses showed that the nucleotide diversity of *rbcL* is relatively low compared to other loci (PI < 0.05) (Fig. 5, Table S1), which helps explain the low support found in phylogenies inferred with this gene [7]. We detected nine hot spots in coding regions and 12 hot spots in noncoding regions, which can be used as candidate DNA barcodes for future studies. These variable regions may also be useful for assessing phylogenetic relationships and interspecific differences of Myrtales species.

### Phylogenetic relationships of Myrtales

Compared with previous studies based on a few chloroplast genome fragments, our results based on the major lineages of Myrtales (six families with more species within Myrtales) showed a highly resolved phylogenetic relationship of Myrtales by using whole chloroplast genomes [2, 6, 28]. Six major clades representing the major families are fully resolved with strong support (Fig. 7). Previous studies of Myrtales have provided an improved understanding of phylogenetic relationships among families based on both morphological and molecular analyses, however, the placement of Combretaceae has not been fully established with high confidence [2, 6, 28]. The phylogenetic location of Combretaceae is critical since its placement directly affects the age of Myrtales, hypotheses of diffusion and variation scenarios, species diversification rates, and features of trait reconstructions [2]. Most recent phylogenetic studies use a limited number of taxa and gene regions as placeholders for Combretaceae [7, 28, 46, 47]. Our plastome phylogenomic analysis of Myrtales provides strong support for the sister relationship between Combretaceae and a clade of Onagraceae + Lythraceae (BS = 100%, PP = 1; Fig. 7), which is in agreement with some previous molecular studies, and a clade of Combretaceae + Onagraceae + Lythraceae is sister to a clade of Melastomataceae + Myrtaceae + Vochysiaceae [18, 48]. The sampling of our study is not comprehensive at the family level with the phylogenetic relationship reconstructed including six of the nine families (lack samples from Crypteroniaceae, Penaeaceae and Alzateaceae). However, according to previous studies, this does not affect our determination of the phylogenetic position of the Combretaceae. We used the whole chloroplast genome to construct the phylogenetic relationships, as well as using multiple chloroplast gene data sets (excluding the chloroplast genome of IRa region, coding genes, noncoding genes, LSC, SSC, IRb) to compare the phylogenetic relationship comprehensively. We also reconstructed the phylogenetic relationship by adding extra taxa (within the branch of Malvids), providing an additional degree of credibility for the obtained phylogenetic trees [49, 50] and determining the phylogenetic position of the Combretaceae. Further research should include sampling more individuals from wild populations and obtaining more extensive nuclear data to determine whether our results are consistent with those from nuclear genes.

### Molecular dating

Biogeography estimates generally suggested that the Myrtales originated in Gondwana [7, 28, 46, 51, 52] with the diversity of all major stem lineages being traced to 85–90 Ma in the western portion of Gondwana. The results of the molecular dating showed that the crown group of Myrtales most likely originated in the Albian age of the Lower Cretaceous [104.90 Ma (95% HPD = 87.88–114.18 Ma)]. The estimated divergence time of Myrtales (Fig. 6) presented here is in close proximity to previously reported dates (104.90 Ma compared to 111 Ma, Sytsma et al. [28]; 116.4 Ma, Berger et al. [2]; 90.7 Ma, Thornhill et al. [53]). However, Gonçalves et al. [54] using 78 protein coding genes from 122 chloroplast genomes of Myrtales, combined with four Myrtales fossil sites and a secondary calibration point, estimated the divergence time of Myrtales to be 125.5 Ma (95% HPD = 130.9–120.3 Ma) during the upper Cretaceous. Fossil limitations, different methods, size of molecular data and taxonomic sampling cannot be perfectly compared across all studies, with changes leading to differences in age estimates. Our analysis estimated that the diversity of major lineages of Myrtales occurred about 60–90 Ma [2, 18]. In this period the species within Myrtales may have begun to differentiate rapidly, which is consistent with the common hypothesis that many species experienced rapid diversification events after the Cretaceous-Paleogene (K-Pg) boundary due to mass extinction and opening of new habitats [55–57]. Our results show that the species diversity of the main stem lineages of Myrtales increased at the end of the Campanian and may have been affected by the continental breakup of Gondwana in the Cretaceous [2].

## Conclusions

In this study, we analyzed and compared the structural characteristics of chloroplast genomes of Myrtales, and inferred the phylogenetic divergence time of Myrtales. The chloroplast genomes of Myrtales has a typical four part structure, including 77–81 protein coding genes, 29–31 tRNA genes and four rRNA genes, with a total length of 152,214–171,315 bp. We found 21 mutation hotspots, which can be used as potential DNA barcodes in the future phylogenetic study of Myrtales. Phylogenetic relationships (Ml / BI) based on whole chloroplast genome and multiple datasets showed that Myrtales and its families were monophyletic, as well as Combretaceae and Onagraceae + Lythraceae strongly supported as a clade, (BS = 100%, PP = 1). Reconstructing the divergence time of Myrtales shows that the crown of Myrtales is 104.90 Ma (95% HPD = 87.88–114.18 Ma), and it differentiated from Geraniales around 111.59 MA (95% HPD = 95.50–118.62 MA) in the Albian of the early Cretaceous. The species divergence of Myrtales ranged from 60 to 90 Ma. These chloroplast genomes contribute to the study of genetic diversity and species evolution of Myrtales, while providing useful information for taxonomic and phylogenetic studies of Myrtales. In the future, we will expand genomic sampling, including nuclear genomes, to comprehensively compare and discuss the phylogeny and evolution of Myrtales species.

## Methods

### Taxon sampling

Leaf material from nine species, representing seven genera and three families in Myrtales, was collected and stored in silica gel. *Combretum kraussii* Hochst., *Eucalyptus grandis* W. Mill ex Maiden, *Melaleuca leucadendra* Linn., *Combretum littoreum* (Engl.) Exell, *Syzygium forrestii* Merr. et Perry, *S. cumini* (Linn.) Skeels and *Tibouchina semidecandra* Cogn. were collected from the Ruili Botanical Garden (Yunnan Province, China; 23°52′ to 24°09′ E, 97°38′ to 98°05′ N). *Combretum malabaricum* Linn. and *Terminalia catappa* Linn. were collected from Hainan University (Hainan province of China; 20°05′ to 20°06′ E, 110°33′ to 110°34′ N). The sampling of nine newly sequenced species was approved by Ruili Botanical Garden (Yunnan Province, China) and Hainan University (Hainan province of China) and met local policy requirements. Table 3 indicates the detailed voucher and locality information for the newly sequenced species. In addition, 83 species representing six families of Myrtales and three outgroups (*Viviania marifolia*, NC_023259.1; *Pelargonium tetragonum*, NC_031205.1; *Pelargonium quercifolium*, NC_031203.1) were downloaded from NCBI with detailed information presented in Table 1. We also downloaded 17 chloroplast genomes from NCBI, including six different orders to serve as outgroups to construct a branch of Malvids to explore the topological changes of Myrtales (Table S2).

**Table 3:** GenBank access numbers, voucher specimen, location information and reference template for plastome assembly of nine newly sequenced genomes.

Family	Species name	Accession number	Specimen collection and voucher specimen	Locality	Latitude	Longitude	Template for plastome assembly
Melastomataceae	*Tibouchina semidecandra*	MT700492	HCNGB, RL0146	Ruili Botanical Garden, Yunnan Province, China	97°38′47″ to 98°05′57″ N	23°52′42″ to 24°09′20″ E	*Pterogastra divaricata NC_031885.1*
Myrtaceae	*Eucalyptus grandis*	MT700491	HCNGB, RL0106	Ruili Botanical Garden, Yunnan Province, China	97°38′47″ to 98°05′57″ N	23°52′42″ to 24°09′20″ E	*Corymbia tessellaris* *NC_022410.1*
Myrtaceae	*Syzygium forrestii*	MK102721.1	HCNGB, RL0700	Ruili Botanical Garden, Yunnan Province, China	97°38′47″ to 98°05′57″ N	23°52′42″ to 24°09′20″ E	*Acca sellowiana* *KX289887.1*
Myrtaceae	*Syzygium cumini*	MT700494	HCNGB, RL0850	Ruili Botanical Garden, Yunnan Province, China	97°38′47″ to 98°05′57″ N	23°52′42″ to 24°09′20″ E	*Acca sellowiana* *KX289887.1*
Myrtaceae	*Melaleuca leucadendra*	MT700493	HCNGB, RL0233	Ruili Botanical Garden, Yunnan Province, China	97°38′47″ to 98°05′57″ N	23°52′42″ to 24°09′20″ E	*Acca sellowiana* *KX289887.1*
Combretaceae	*Combretum kraussii*	MT700495	HCNGB, RL0855	Ruili Botanical Garden, Yunnan Province, China	97°38′47″ to 98°05′57″ N	23°52′42″ to 24°09′20″ E	*Lagerstroemia speciosa* *KX572149.1*
Combretaceae	*Combretum littoreum*	MT700496	HCNGB, RL0942	Ruili Botanical Garden, Yunnan Province, China	97°38′47″ to 98°05′57″ N	23°52′42″ to 24°09′20″ E	*Eucalyptus grandis* *HM347959.1*
Combretaceae	*Terminalia catappa*	MT700489	B244	Hainan University in Hainan province of China	110°33′ 41″to 110°34′17″ N	20°05′38″ to 20°06′ 23″E	*Eucalyptus grandis* *HM347959.1*
Combretaceae	*Combretum malabaricum*	MT700490	B246	Hainan University in Hainan province of China	110°33′ 41″to 110°34′18″ N	20°05′38″ to 20°06′ 24″E	*Eucalyptus grandis* *HM347959.1*

*HCNGB* Herbarium of China National GenBank, *HUTB* Herbarium of the Institute of Tropical Agriculture and Forestry, Hainan University

### DNA extraction, sequencing and assembly

We used a modified cetyltrimethyl ammonium bromide (CTAB) method to extract high quality DNA from dried leaves [58]. Quality of DNA was determined on an Agilent 2100 BioAnalyzer by using ≥0.8 μg at the University of California Davis Genome Center (Davis, California, USA). We constructed paired-end sequencing libraries with insert sizes of 200–400 bp with Illumina TruSeq™ Nano DNA Sample Prep Kit and sequenced using the BGISEQ-500 at the Beijing Genomics Institution (BGI; Shenzhen, China). Raw reads were filtered with SOAPfilter_v2.2 for quality control with the following parameters: 1) remove low quality reads (> 10% Ns and/or > 40% low quality bases), 2) remove PCR duplicates, and 3) trim adaptor sequences. We selected the *rbcL* gene of *Arabidopsis thaliana* from NCBI (accession number: U91966) as a seed and assembled chloroplast genomes for each species using the clean reads with NOVOPlasty [59]. The longest contig assembled by NOVOPlasty was compared with chloroplasts deposited in the NCBI database, and obtained the chloroplast genome sequence with the highest homology (minimum requirement: e-value < 10–7, identity > 95%) to us as the reference (Table 3) for subsequent assembly using MITObim v1.8 [60]. Quality of the assemblies were assessed by mapping clean reads using BWA MEM (Burrows-Wheeler Aligner) v0.7.17 [61] to verify the integrity of newly assembled plastome [62].

### Plastome annotation

Plastome sequences were initially annotated using Geneious R11.0.4 (Biomatters Ltd., Auckland, New Zealand), then further annotated with Dual Organellar GenoMe Annotator (DOGMA) [63] to modify gene boundaries. The tRNA genes were verified with tRNAscan-SE1.21 [64]. Maps were drawn using OrganellarGenomeDRAW v1.3.1 (available online: https://chlorobox.mpimp-golm.mpg.de/OGDraw.html) [65] (Fig. 3). All plastome sequences have been uploaded to NCBI (Table 3).

### Plastome comparative analysis and molecular marker identification

Plastome comparisons across 24 Myrtales species (15 samples from NCBI and the nine newly sequenced chloroplast genomes covering 16 subfamilies/families within Myrtales) were performed in Shuffle-LAGAN mode on the mVISTA program (genome.lbl.gov/vista/index.shtml [66];), using the annotation of *Vochysia acuminate* (NC_043811) as a reference. To reveal highly variable regions for future species identification studies and to evaluate different plastome regions that may show different evolutionary patterns, we sequentially extracted both coding regions and noncoding regions (including intergenic spacers and introns) after alignment with MAFFT v7 [67] using the criteria that the aligned length is > 200 bp and at least one mutation per site was present. The nucleotide variability of the selected regions was evaluated using DNASP v5.10 [68]. The IR / SC boundary map of these 24 Myrtales chloroplast was drawn with Photoshop. The IR area was confirmed using UNIPRO ugene v1.32 [69].

### Phylogenetic analysis

Phylogenetic analyses were conducted on 95 species, using *Viviania marifolia* (NC_023259), *Pelargonium tetragonum* (NC_031205), and *Pelargonium quercifolium* (NC_031203) as outgroups based on a previous study [2]. Plastome sequences were aligned using MAFFT v7 [67] and manually checked when necessary. The complete chloroplast genome sequence and chloroplast genome minus one copy of the inverted repeat (No-IRa) were used to construct the phylogenetic topology using maximum likelihood (ML) and Bayesian inference (BI). To evaluate alternative hypotheses, phylogenetic topologies were inferred using both maximum likelihood (ML) and Bayesian inference (BI) methods using the complete plastome sequences and whole plastome minus one copy of the Inverted Repeat (No-IRa). We also included other data sets (i.e., coding area, noncoding area, LSC, SSC and IRb) for analyses. The best-fitting model of molecular evolution (GTR + GAMMA+I) (Table 4) was determined using Akaike Information Criterion (AIC) in JMODELTEST v2.1.7 [70]. Maximum likelihood analyses were conducted in RAxML-HPC v8.2.8 [71] with 1000 bootstrap replicates on the CIPRES Science Gateway portal [72]. Bayesian analyses were performed in MRBAYES v3.2 [73]. Two independent Markov Chain Monte Carlo chains were conducted simultaneously for 5 million generations with trees sampled every 1000 generations. The effective sample size (ESS > 200) was determined using Tracer v1.7 [74] and the first 25% of trees were discarded as burn-in, and a consensus tree was constructed from the remaining trees to estimate posterior probabilities (PPs). FigTree v1.4.4 [75] were used for visualizing the resulting phylogenetic trees.

**Table 4:** Characteristics and models selected in ML and BI phylogenetic analyses with different subsets of data

Datasets	Number of taxa	Number of sites	Number of variable/Parsimony informative sites	Best fit Model	Model in ML	Model in BI
Whole plastid genomes	95	130,398	57,674/38001	GTR + I + G	GTR + G	GTR + I + G
Coding	95	71,672	28,966/19612	GTR + I + G	GTR + G	GTR + I + G
Non-coding	95	107,087	52,927/34467	GTR + I + G	GTR + G	GTR + I + G
IRb	95	66,767	24,634/10015	TVM + G	GTR + G	TVM + G
LSC	95	181,032	90,119/49802	TVM + I + G	GTR + G	TVM + I + G
SSC	95	34,453	19,663/12511	GTR + G	GTR + G	GTR + G
NON-IRa	95	251,669	103,802/65656	GTR + G	GTR + G	GTR + G

### Divergence time estimation

The complete 92 plastome dataset of Myrtales was analyzed using the GTR + GAMMA+I model selected by MrModelTest [76] in BEAST v.1.8.4 [75] to simultaneously search for the best tree topology and estimate node ages. The divergence time between lineages was estimated using a Yule speciation prior and an uncorrelated lognormal model of rate change with a relaxed clock. Four fossil-based calibration points and one secondary calibration point were used to constrain the crown node age of Myrtales. (1) The Myrtaceidites (=Syncolporites) pollen [28] placed a prior on the crown of Myrtaceae. The *Myrtaceidites lisamae* (83.5 Ma) fossil from Gabon, Africa during the Santonian [52, 77, 78] was considered the oldest fossil in Myrtaceae. Therefore, we set the stem of Myrtaceae with a lognormal mean = 0, a SD = 1.0 and an offset = 83.5 Ma. (2) In the Chamelaucioideae clade of Myrtaceae we placed the fossil of *Eucalyptus frenguelliana* (51.69 Ma) dated to the early Eocene from Laguna del Hunco in Chubut Province, Argentina [79, 80]. We set the stem of Chamelaucioideae with a lognormal mean = 0, a SD = 1.0 and an offset = 51.69 Ma. (3) The stem of Lythraceae was set to a lognormal mean = 0, a SD = 1.0 and an offset = 81.0 Ma based on the pollen fossil for *Lythrum elkensis* of Lythrum/Peplis from the Late Cretaceous (early Campanian, 82–81 Ma) in Wyoming, USA [80, 81]. (4) We used the earliest recorded wood fossil of *Sonneratioxylon preapetalum* Awasthi [82] from the early Paleocene in India (Danian, 67.3–63.8 Ma) [81] to constrain the node of Trapoideae. We set the stem to 63.8 Ma with a lognormal mean equal to 0 and a standard deviation of 1. (5) Based on the results of Li et al. [18], the clade of Myrtales and Cerambycidales had a crown age of 112.26 Ma, the crown node age of Myrtales+Geraniales was constrained to 112.26 Ma, with a normal prior and SD = 5. Nine runs each with 100 million generations were conducted totaling 900 million generations with parameters sampled every 1000 generations. The effective sample size (> 200) was determined using Tracer v1.6 [75] and the first 25% of the samples were discarded as burn-in. TreeAnnotator v1.8.0 [75] was used to produce a maximum clade credibility chronogram showing the mean divergence time estimates with 95% highest posterior density (HPD) intervals. FigTree v1.4.4 [75] was used to visualize the resulting divergence times.

## Supplementary Information

**Additional file 1: Figures S1–S6.** are phylogenetic relationships inferred by Maximum Likelihood and Bayesian inference based on: coding genes; noncoding loci; the LSC (the Large Single-Copy); the SSC (the Small Single-Copy); NO-IRa data set (data set composition is described in the methods) and IRb (Inverted Repeat region). Support values are maximum likelihood bootstrap support/Bayesian posterior probability. The families of Myrtales are indicated by different colors. For each figure, the inset shows the same tree as a phylogram (except for some inconsistencies in the phylogenetic relationships of IR dataset construction). The support value on the branch is bootstrap value/Bayesian posterior probability: “*” means 100% /1.0 support value, and “-” means bootstrap value/Bayesian posterior probability is less than 60 / 0.7. The families of Myrtales are represented by different colors. The small picture in the upper left corner is the ML phylogenetic tree (showing branch length).

**Additional file 2: Figure S7.** Optimal phylogenetic tree resulting from analyses of 92 complete chloroplast genomes of Myrtales and 20 outgroups using Maximum Likelihood (ML). Support values are maximum likelihood bootstrap support posterior probability. The families of Myrtales are indicated by different colors. . The support value on the branch is bootstrap value, “*” means 100% support value, and “-” means bootstrap value is less than 60. The families of Myrtales are represented by different colors. The small picture in the upper left corner is the ML phylogenetic tree (showing branch length).

**Additional file 3: Table S1.** Eta, Pi value, H, Hd, PICs, the length and aligned length of 188 Myrtales homologous loci across.

**Additional file 4: Table S2.** Species information and chloroplast genomes GenBank accession number of Outgroups in this study.

## Data Availability

All sequences used in this study are available from the National Center for Biotechnology Information (NCBI) (accession numbers: MT700492- MT700490; see Additional Table 2).

## Abbreviations

BI: Bayesian Inference
CTAB: Cetyltrimethylammonium bromide
DnaSP: DNA Sequences Polymorphism
IR: Inverted repeat
LSC: Large single copy
GTR: General time reversible
ML: Maximum Likelihood
PI: Phylogenetic informativeness
rRNA: Ribosomal RNA
SSC: Small single copy
tRNA: Transfer RNA
