# Supplementary figures and images for: Comparative analysis of chloroplast genome structure and molecular dating in Myrtales
Source: BMC Plant Biol. 2021 May 15;21:219. doi: 10.1186/s12870-021-02985-9 (PMC8122561; doi:10.1186/s12870-021-02985-9)

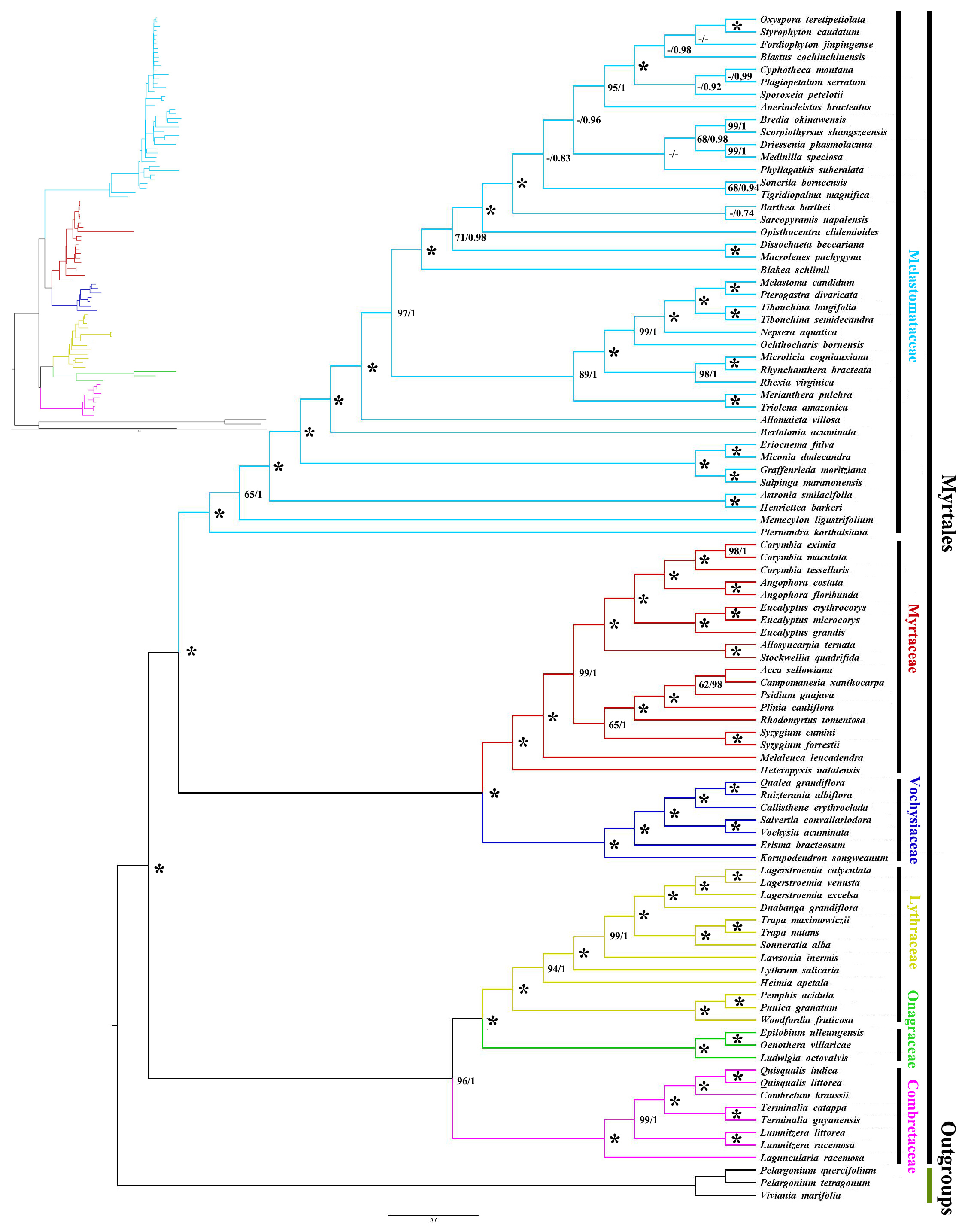

Supplement: Supplementary file 1 — Additional file 1: Figures S1–S6. are phylogenetic relationships inferred by Maximum Likelihood and Bayesian inference based on: coding genes; noncoding loci; the LSC (the Large Single-Copy); the SSC (the Small Single-Copy); NO-IRa data set (data set composition is described in the methods) and IRb (Inverted Repeat region). Support values are maximum likelihood bootstrap support/Bayesian posterior probability. The families of Myrtales are indicated by different colors. For each figure, the inset shows the same tree as a phylogram (except for some inconsistencies in the phylogenetic relationships of IR dataset construction). The support value on the branch is bootstrap value/Bayesian posterior probability: “*” means 100% /1.0 support value, and “-” means bootstrap value/Bayesian posterior probability is less than 60 / 0.7. The families of Myrtales are represented by different colors. The small picture in the upper left corner is the ML phylogenetic tree (showing branch length). [file 12870_2021_2985_MOESM1_ESM.zip › Fig.S1.jpg]

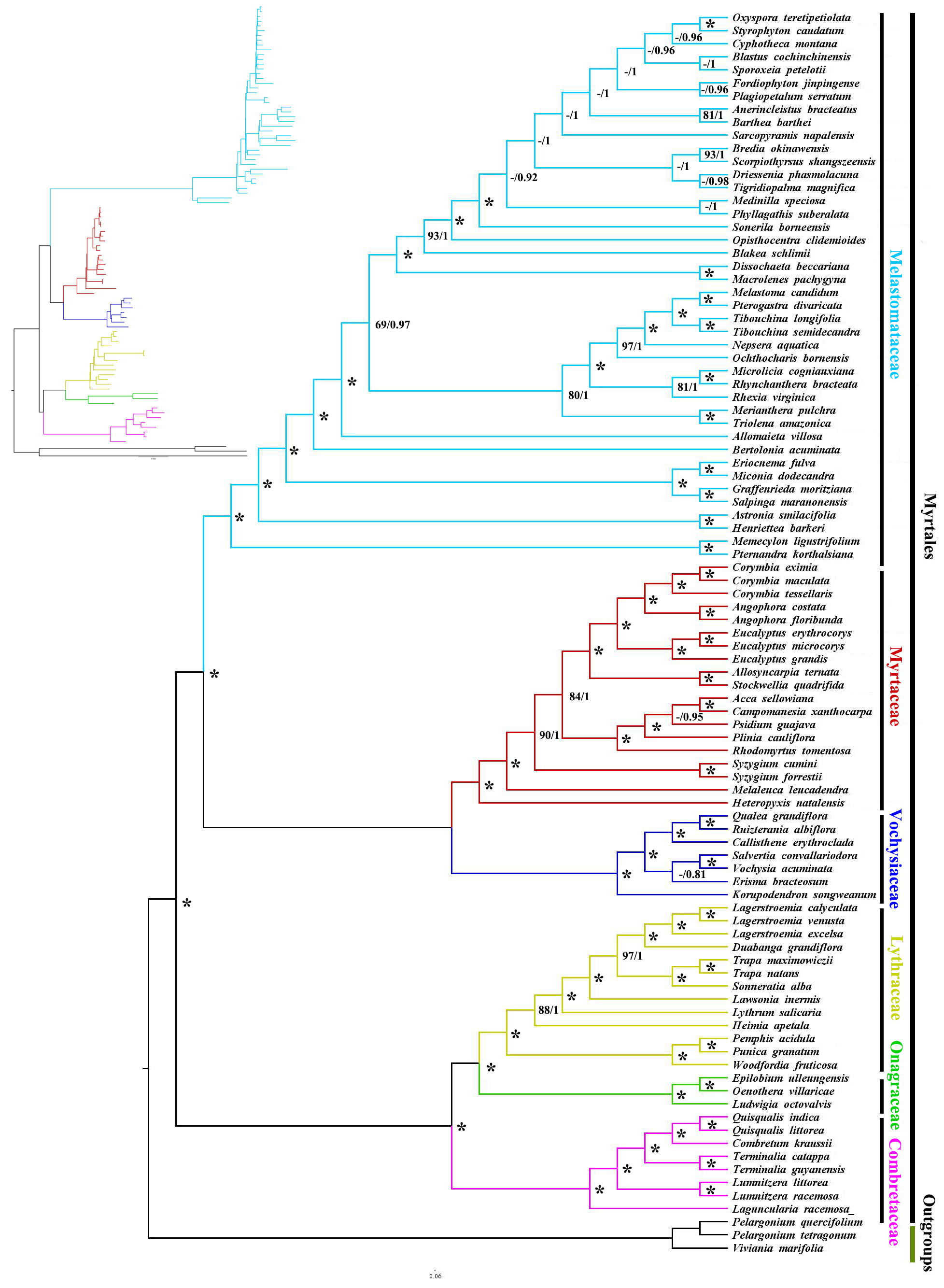

Supplement: Supplementary file 1 — Additional file 1: Figures S1–S6. are phylogenetic relationships inferred by Maximum Likelihood and Bayesian inference based on: coding genes; noncoding loci; the LSC (the Large Single-Copy); the SSC (the Small Single-Copy); NO-IRa data set (data set composition is described in the methods) and IRb (Inverted Repeat region). Support values are maximum likelihood bootstrap support/Bayesian posterior probability. The families of Myrtales are indicated by different colors. For each figure, the inset shows the same tree as a phylogram (except for some inconsistencies in the phylogenetic relationships of IR dataset construction). The support value on the branch is bootstrap value/Bayesian posterior probability: “*” means 100% /1.0 support value, and “-” means bootstrap value/Bayesian posterior probability is less than 60 / 0.7. The families of Myrtales are represented by different colors. The small picture in the upper left corner is the ML phylogenetic tree (showing branch length). [file 12870_2021_2985_MOESM1_ESM.zip › Fig.S2.jpg]

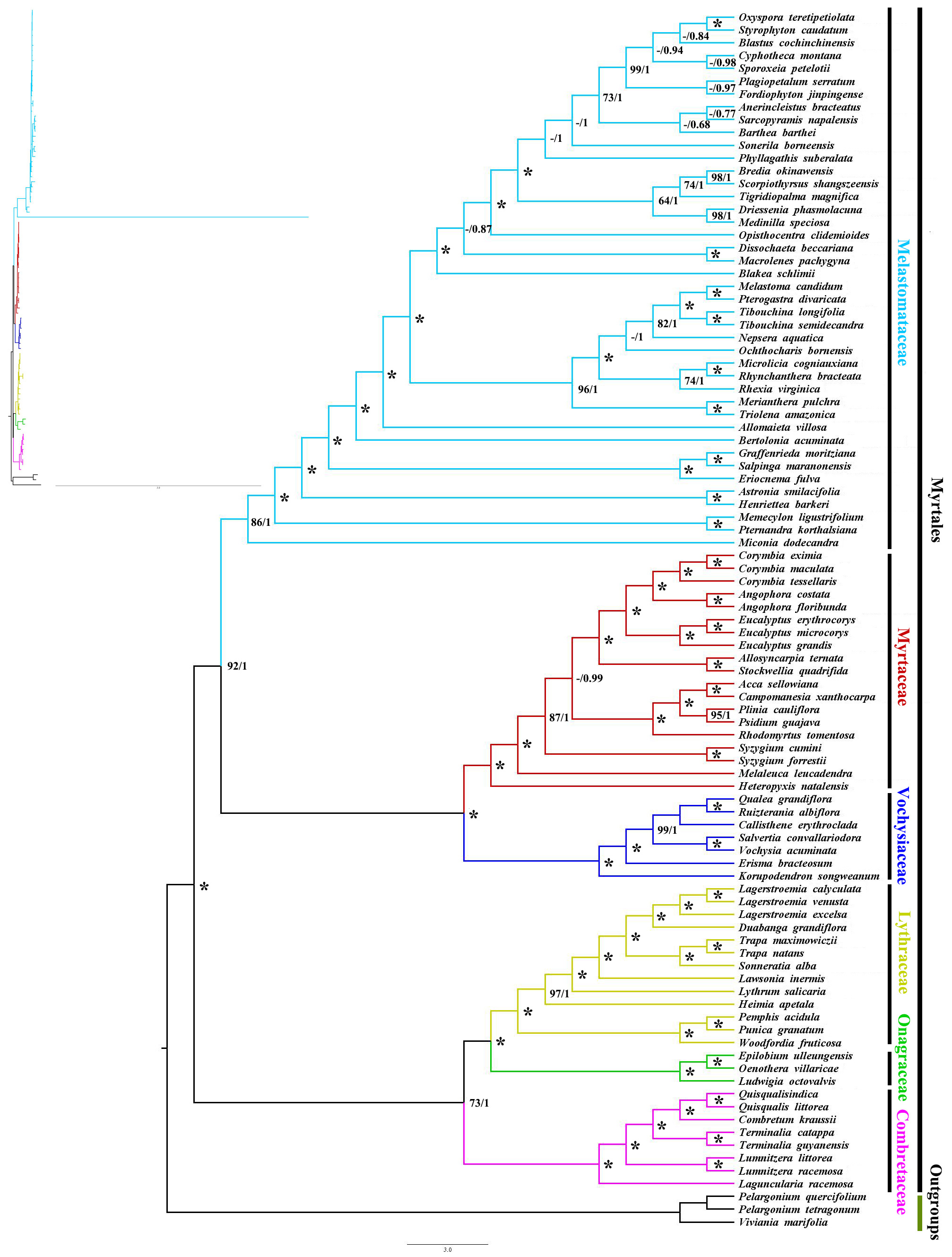

Supplement: Supplementary file 1 — Additional file 1: Figures S1–S6. are phylogenetic relationships inferred by Maximum Likelihood and Bayesian inference based on: coding genes; noncoding loci; the LSC (the Large Single-Copy); the SSC (the Small Single-Copy); NO-IRa data set (data set composition is described in the methods) and IRb (Inverted Repeat region). Support values are maximum likelihood bootstrap support/Bayesian posterior probability. The families of Myrtales are indicated by different colors. For each figure, the inset shows the same tree as a phylogram (except for some inconsistencies in the phylogenetic relationships of IR dataset construction). The support value on the branch is bootstrap value/Bayesian posterior probability: “*” means 100% /1.0 support value, and “-” means bootstrap value/Bayesian posterior probability is less than 60 / 0.7. The families of Myrtales are represented by different colors. The small picture in the upper left corner is the ML phylogenetic tree (showing branch length). [file 12870_2021_2985_MOESM1_ESM.zip › Fig.S3.jpg]

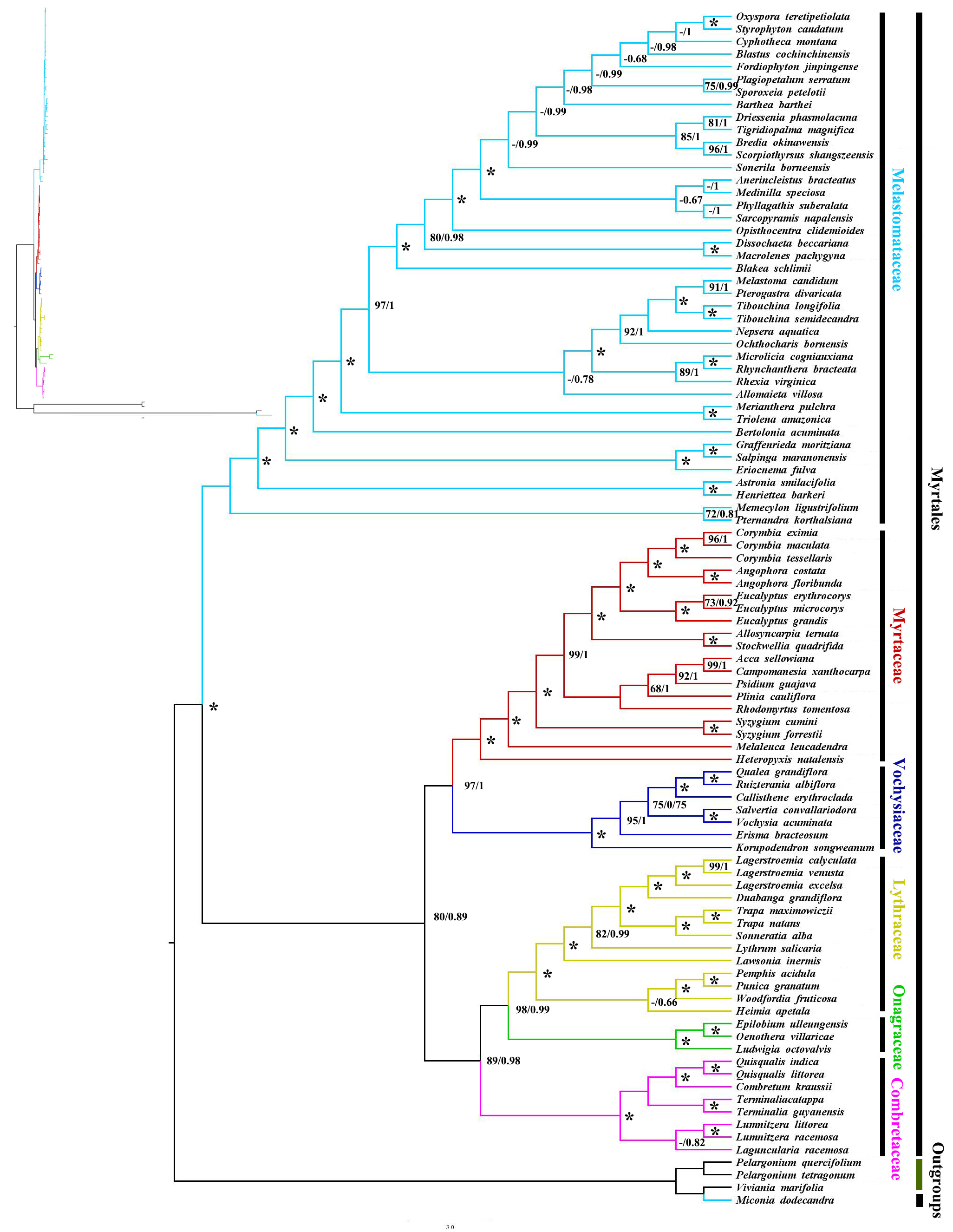

Supplement: Supplementary file 1 — Additional file 1: Figures S1–S6. are phylogenetic relationships inferred by Maximum Likelihood and Bayesian inference based on: coding genes; noncoding loci; the LSC (the Large Single-Copy); the SSC (the Small Single-Copy); NO-IRa data set (data set composition is described in the methods) and IRb (Inverted Repeat region). Support values are maximum likelihood bootstrap support/Bayesian posterior probability. The families of Myrtales are indicated by different colors. For each figure, the inset shows the same tree as a phylogram (except for some inconsistencies in the phylogenetic relationships of IR dataset construction). The support value on the branch is bootstrap value/Bayesian posterior probability: “*” means 100% /1.0 support value, and “-” means bootstrap value/Bayesian posterior probability is less than 60 / 0.7. The families of Myrtales are represented by different colors. The small picture in the upper left corner is the ML phylogenetic tree (showing branch length). [file 12870_2021_2985_MOESM1_ESM.zip › Fig.S4.jpg]

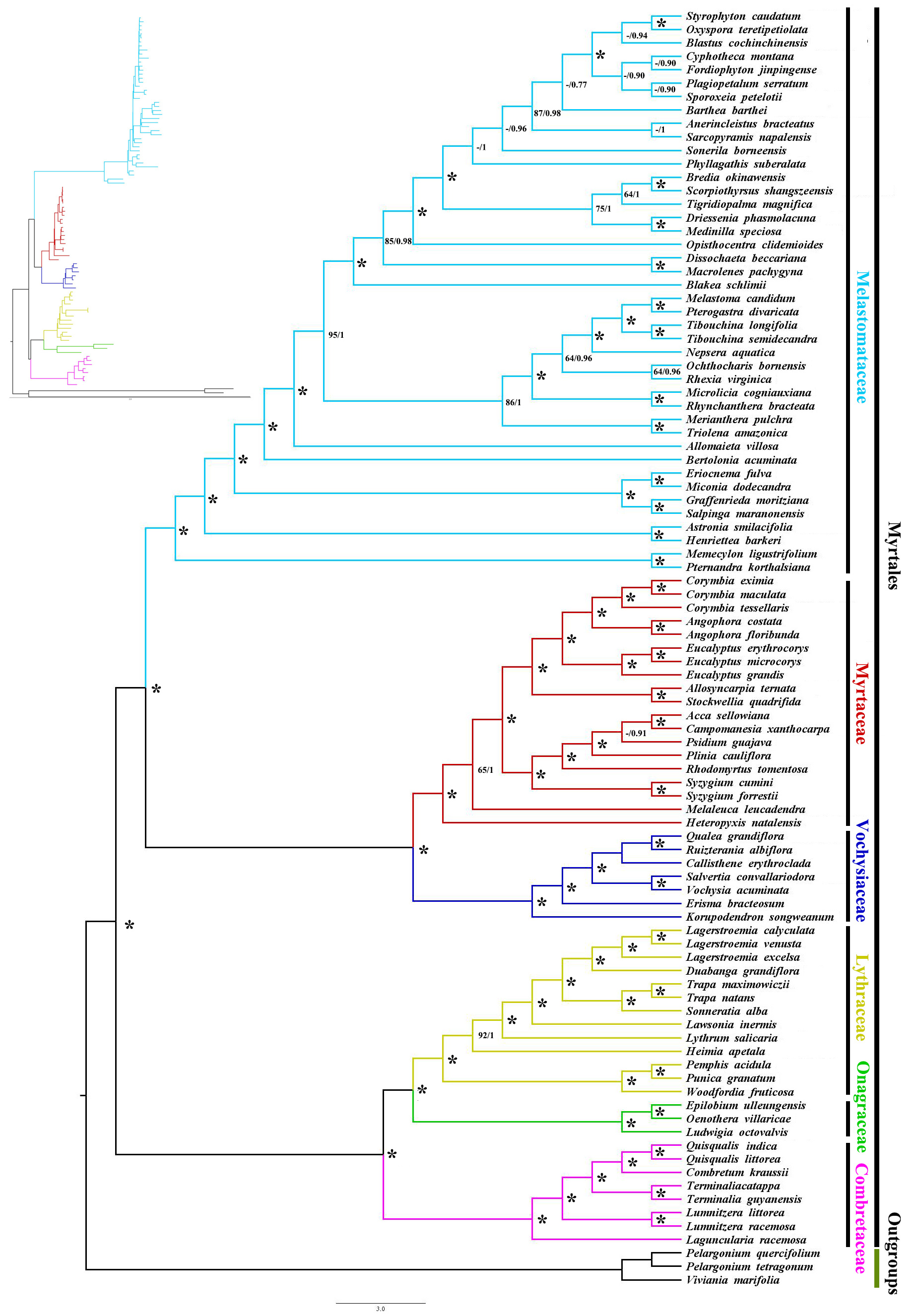

Supplement: Supplementary file 1 — Additional file 1: Figures S1–S6. are phylogenetic relationships inferred by Maximum Likelihood and Bayesian inference based on: coding genes; noncoding loci; the LSC (the Large Single-Copy); the SSC (the Small Single-Copy); NO-IRa data set (data set composition is described in the methods) and IRb (Inverted Repeat region). Support values are maximum likelihood bootstrap support/Bayesian posterior probability. The families of Myrtales are indicated by different colors. For each figure, the inset shows the same tree as a phylogram (except for some inconsistencies in the phylogenetic relationships of IR dataset construction). The support value on the branch is bootstrap value/Bayesian posterior probability: “*” means 100% /1.0 support value, and “-” means bootstrap value/Bayesian posterior probability is less than 60 / 0.7. The families of Myrtales are represented by different colors. The small picture in the upper left corner is the ML phylogenetic tree (showing branch length). [file 12870_2021_2985_MOESM1_ESM.zip › Fig.S5.jpg]

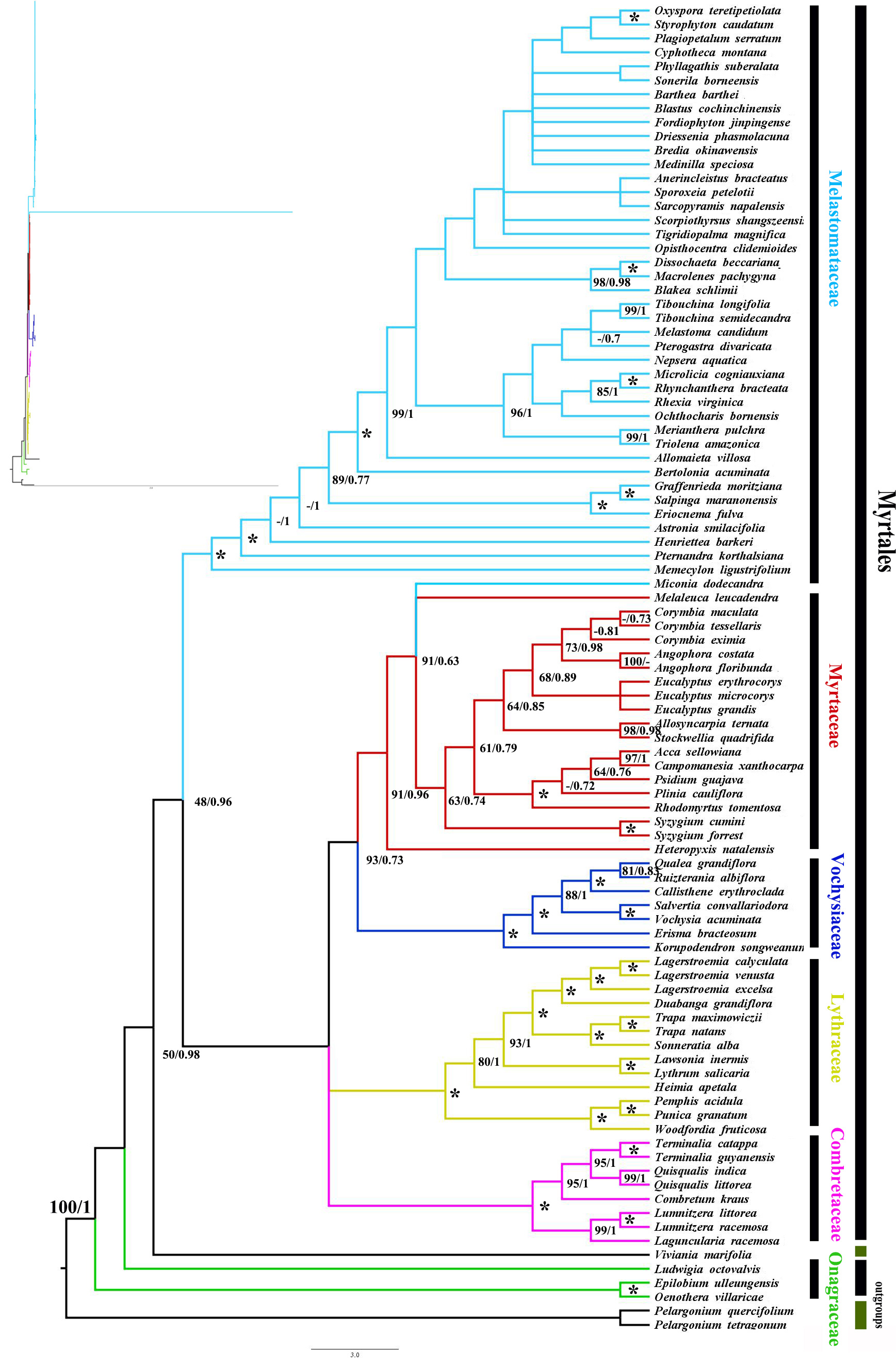

Supplement: Supplementary file 1 — Additional file 1: Figures S1–S6. are phylogenetic relationships inferred by Maximum Likelihood and Bayesian inference based on: coding genes; noncoding loci; the LSC (the Large Single-Copy); the SSC (the Small Single-Copy); NO-IRa data set (data set composition is described in the methods) and IRb (Inverted Repeat region). Support values are maximum likelihood bootstrap support/Bayesian posterior probability. The families of Myrtales are indicated by different colors. For each figure, the inset shows the same tree as a phylogram (except for some inconsistencies in the phylogenetic relationships of IR dataset construction). The support value on the branch is bootstrap value/Bayesian posterior probability: “*” means 100% /1.0 support value, and “-” means bootstrap value/Bayesian posterior probability is less than 60 / 0.7. The families of Myrtales are represented by different colors. The small picture in the upper left corner is the ML phylogenetic tree (showing branch length). [file 12870_2021_2985_MOESM1_ESM.zip › Fig.S6.jpg]

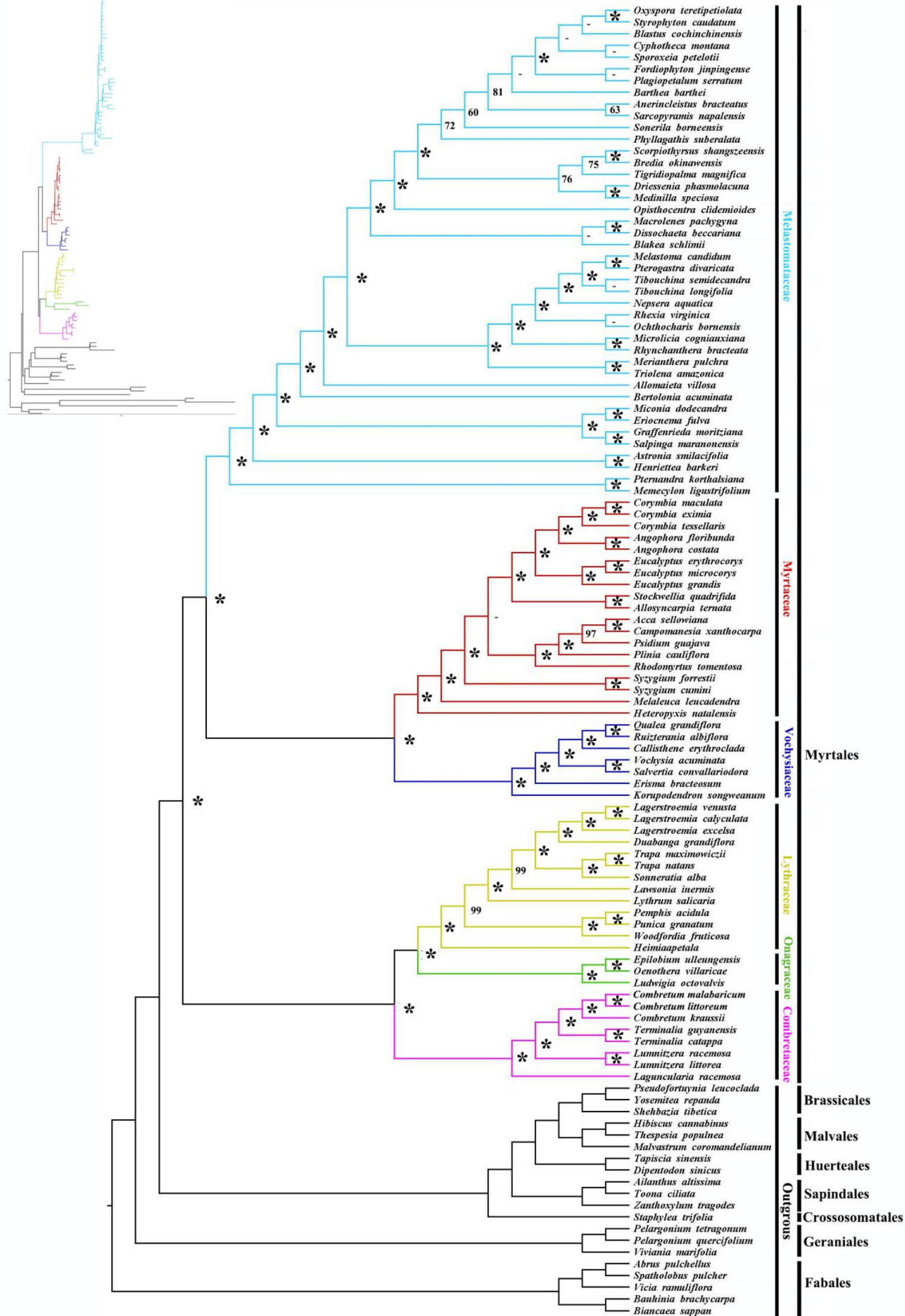

Supplement: Supplementary file 2 — Additional file 2: Figure S7. Optimal phylogenetic tree resulting from analyses of 92 complete chloroplast genomes of Myrtales and 20 outgroups using Maximum Likelihood (ML). Support values are maximum likelihood bootstrap support posterior probability. The families of Myrtales are indicated by different colors. . The support value on the branch is bootstrap value, “*” means 100% support value, and “-” means bootstrap value is less than 60. The families of Myrtales are represented by different colors. The small picture in the upper left corner is the ML phylogenetic tree (showing branch length). [file 12870_2021_2985_MOESM2_ESM.pdf]
