# Supplementary material for: Comparative analysis of chloroplast genome structure and molecular dating in Myrtales
Source: BMC Plant Biol. 2021 May 15;21:219. doi: 10.1186/s12870-021-02985-9 (PMC8122561; doi:10.1186/s12870-021-02985-9)
Supplement: Supplementary file 3 — Additional file 3: Table S1. Eta, Pi value, H, Hd, PICs, the length and aligned length of 188 Myrtales homologous loci across. [file 12870_2021_2985_MOESM3_ESM.docx]

Eta, Pi value, H,Hd,PICs,the length and aligned length of 188 Myrtales homologous loci across

| **Region** | **Total number of mutation (Eta)** | **Divergence variation (Pi)** | **Number of Haplotypes(h)** | **Haplotype (gene) diversity**（Hd） | **Parsimony informative sites** | **Length (bp)** | **aligned lenght (bp)** | **Number of sequences** |
| --- | --- | --- | --- | --- | --- | --- | --- | --- |
| **Coding gene** | | | | | | | | |
| accD | 839 | 0.07894 | 90 | 1 | 359 | 1362-1641 | 2615 | 92 |
| atpA | 603 | 0.05584 | 87 | 0.998 | 323 | 1506-1548 | 1548 | 92 |
| atpB | 390 | 0.0439 | 85 | 0.997 | 204 | 1170-1500 | 1501 | 92 |
| atpE | 181 | 0.05665 | 78 | 0.995 | 93 | 399-423 | 423 | 92 |
| atpF | 282 | 0.06584 | 83 | 0.996 | 144 | 507-633 | 633 | 92 |
| atpH | 144 | 0.04108 | 60 | 0.973 | 35 | 246 | 246 | 92 |
| atpI | 251 | 0.0437 | 79 | 0.994 | 138 | 744-750 | 753 | 92 |
| ccsA | 560 | 0.0889 | 87 | 0.998 | 266 | 885-966 | 1017 | 92 |
| cemA | 331 | 0.0649 | 82 | 0.996 | 166 | 651-699 | 723 | 92 |
| clpP | 409 | 0.05548 | 73 | 0.989 | 182 | 573-759 | 804 | 92 |
| matK | 747 | 0.12578 | 89 | 0.999 | 375 | 882-1590 | 1800 | 92 |
| ndhA | 523 | 0.06722 | 88 | 0.999 | 257 | 1071-1104 | 1107 | 92 |
| ndhB | 146 | 0.01117 | 62 | 0.983 | 74 | 1503-1548 | 1563 | 92 |
| ndhC | 71 | 0.03522 | 60 | 0.98 | 37 | 213-363 | 363 | 92 |
| ndhD | 867 | 0.07947 | 90 | 1 | 433 | 1479-1539 | 1564 | 92 |
| ndhE | 120 | 0.05138 | 80 | 0.996 | 68 | 304-306 | 306 | 92 |
| ndhF | 948 | 0.13053 | 90 | 1 | 424 | 1077-2376 | 2576 | 92 |
| ndhG | 277 | 0.06785 | 83 | 0.997 | 130 | 529-531 | 531 | 92 |
| ndhH | 513 | 0.05724 | 88 | 0.999 | 263 | 1182 | 1182 | 92 |
| ndhI | 219 | 0.05691 | 85 | 0.997 | 112 | 486-524 | 540 | 92 |
| ndhJ | 196 | 0.05956 | 79 | 0.993 | 104 | 477 | 477 | 92 |
| ndhK | 311 | 0.05703 | 80 | 0.996 | 151 | 672-873 | 945 | 92 |
| petA | 444 | 0.06526 | 87 | 0.998 | 239 | 957-978 | 981 | 92 |
| petB | 190 | 0.03595 | 78 | 0.993 | 98 | 648-687 | 699 | 92 |
| petD | 138 | 0.04431 | 76 | 0.993 | 82 | 477-540 | 561 | 92 |
| petG | 24 | 0.02329 | 30 | 0.885 | 14 | 114 | 114 | 92 |
| petL | 36 | 0.05785 | 37 | 0.894 | 21 | 96-98 | 98 | 92 |
| petN | 25 | 0.05141 | 29 | 0.906 | 14 | 90-96 | 96 | 92 |
| psaA | 611 | 0.0328 | 89 | 0.999 | 361 | 2252 | 2253 | 92 |
| psaB | 629 | 0.03949 | 87 | 0.999 | 341 | 2151-2205 | 2205 | 92 |
| psaC | 79 | 0.04517 | 60 | 0.978 | 42 | 246 | 246 | 92 |
| psaI | 44 | 0.05603 | 42 | 0.956 | 23 | 105-180 | 195 | 92 |
| psaJ | 58 | 0.0563 | 54 | 0.956 | 28 | 129-141 | 141 | 92 |
| psbA | 268 | 0.03026 | 85 | 0.998 | 150 | 1062 | 1062 | 92 |
| psbB | 543 | 0.04524 | 89 | 0.999 | 270 | 1527 | 1527 | 92 |
| psbC | 416 | 0.03823 | 83 | 0.997 | 221 | 1386-1464 | 1481 | 92 |
| psbD | 236 | 0.0254 | 77 | 0.991 | 137 | 1062 | 1062 | 92 |
| psbE | 54 | 0.03258 | 52 | 0.974 | 32 | 252 | 252 | 92 |
| psbF | 21 | 0.02481 | 25 | 0.874 | 10 | 120 | 120 | 92 |
| psbH | 87 | 0.05539 | 57 | 0.961 | 48 | 222-231 | 242 | 92 |
| psbI | 36 | 0.04351 | 49 | 0.963 | 22 | 111-165 | 165 | 92 |
| psbJ | 39 | 0.02952 | 45 | 0.928 | 15 | 123 | 123 | 92 |
| psbK | 89 | 0.06797 | 65 | 0.979 | 51 | 180-198 | 210 | 92 |
| psbL | 21 | 0.03073 | 25 | 0.889 | 12 | 117 | 117 | 92 |
| psbM | 31 | 0.03838 | 35 | 0.859 | 15 | 105 | 114 | 92 |
| psbN | 27 | 0.01824 | 31 | 0.872 | 13 | 132-270 | 270 | 92 |
| psbT | 31 | 0.02815 | 32 | 0.879 | 13 | 102-117 | 117 | 92 |
| psbZ | 62 | 0.03442 | 55 | 0.965 | 27 | 189 | 189 | 92 |
| rbcL | 469 | 0.04198 | 90 | 1 | 247 | 1428-1458 | 1473 | 92 |
| rpl14 | 162 | 0.0575 | 74 | 0.989 | 88 | 369 | 369 | 92 |
| rpl16 | 213 | 0.06727 | 80 | 0.994 | 107 | 408-432 | 447 | 92 |
| rpl2 | 107 | 0.01533 | 62 | 0.968 | 55 | 732-832 | 841 | 92 |
| rpl20 | 222 | 0.07951 | 82 | 0.994 | 109 | 342-462 | 468 | 92 |
| rpl23 | 66 | 0.02235 | 39 | 0.925 | 30 | 277-309 | 340 | 92 |
| rpl32 | 109 | 0.09787 | 70 | 0.986 | 51 | 153-177 | 262 | 92 |
| rpl33 | 128 | 0.07639 | 73 | 0.992 | 69 | 201-207 | 222 | 92 |
| rpl36 | 74 | 0.05793 | 54 | 0.969 | 29 | 114-147 | 172 | 92 |
| rpoA | 574 | 0.07186 | 88 | 0.998 | 287 | 978-1101 | 1329 | 92 |
| rpoB | 1408 | 0.04838 | 91 | 1 | 703 | 3210-3225 | 3345 | 92 |
| rpoC1 | 632 | 0.04691 | 87 | 0.998 | 309 | 1650-2076 | 2292 | 92 |
| rpoC2 | 2351 | 0.07862 | 90 | 0.07862 | 1174 | 4083-4192 | 4644 | 92 |
| rps11 | 180 | 0.07265 | 72 | 0.991 | 103 | 345-429 | 438 | 92 |
| rps12 | 33 | 0.01921 | 23 | 0.835 | 22 | 351-372 | 473 | 92 |
| rps14 | 137 | 0.04358 | 72 | 0.986 | 60 | 300-303 | 303 | 92 |
| rps15 | 130 | 0.11028 | 74 | 0.993 | 66 | 234-288 | 321 | 92 |
| rps18 | 145 | 0.05409 | 70 | 0.984 | 70 | 300-324 | 657 | 92 |
| rps19 | 129 | 0.08541 | 76 | 0.993 | 65 | 189-294 | 395 | 92 |
| rps2 | 337 | 0.05506 | 85 | 0.998 | 170 | 702-714 | 784 | 92 |
| rps4 | 278 | 0.05489 | 84 | 0.997 | 133 | 606-612 | 690 | 92 |
| rps7 | 50 | 0.00971 | 34 | 0.915 | 23 | 468 | 468 | 92 |
| rps8 | 254 | 0.07224 | 80 | 0.994 | 134 | 405-417 | 417 | 92 |
| ycf1 | 465 | 0.04843 | 61 | 0.977 | 146 | 1138-7203 | 11142 | 92 |
| ycf3 | 131 | 0.03948 | 72 | 0.987 | 73 | 504-513 | 516 | 92 |
| ycf4 | 299 | 0.06262 | 86 | 0.999 | 12 | 555-564 | 564 | 92 |
| **Non-coding regions** | | | | | | | | |
| accD-psaI | 23 | 0.22914 | 24 | 0.836 | 9 | 497-976 | 2182 | 92 |
| atpA-atpF | 45 | 0.11824 | 45 | 0.933 | 19 | 51-86 | 151 | 92 |
| atpB-rbcL | 302 | 0.10806 | 81 | 0.997 | 152 | 731-884 | 1519 | 92 |
| atpF-atpH | 55 | 0.13669 | 45 | 0.924 | 22 | 310-676 | 1238 | 92 |
| atpFintron | 385 | 0.10475 | 84 | 0.997 | 188 | 690-906 | 1386 | 92 |
| atpH-atpI | 222 | 0.14505 | 75 | 0.988 | 111 | 638-1673 | 2916 | 92 |
| atpI-rps2 | 123 | 0.12005 | 71 | 0.987 | 60 | 167-255 | 376 | 92 |
| ccsA-ndhD | 94 | 0.15179 | 65 | 0.97 | 44 | 250-443 | 790 | 92 |
| cemA-petA | 118 | 0.11538 | 71 | 0.99 | 57 | 169-237 | 379 | 92 |
| clpP-psbB | 259 | 0.08996 | 83 | 0.998 | 130 | 393-490 | 747 | 92 |
| matK-trnK(UUU) | 447 | 0.10354 | 89 | 0.999 | 222 | 690-791 | 1211 | 92 |
| ndhAintron | 607 | 0.10894 | 87 | 0.999 | 290 | 940-1161 | 1832 | 92 |
| ndhBintron | 86 | 0.01631 | 50 | 0.952 | 47 | 667-694 | 715 | 92 |
| ndhB-rps7 | 25 | 0.04068 | 17 | 0.726 | 13 | 154-355 | 407 | 92 |
| ndhC-trnV(UAC) | 53 | 0.10918 | 47 | 0.962 | 22 | 424-1499 | 2611 | 92 |
| ndhD-psaC | 58 | 0.14997 | 55 | 0.968 | 29 | 104-194 | 283 | 92 |
| ndhE-ndhG | 145 | 0.18045 | 77 | 0.994 | 73 | 186-256 | 380 | 92 |
| ndhF-rpl32 | 29 | 0.12899 | 26 | 0.854 | 10 | 277-1074 | 2128 | 92 |
| ndhG-ndhI | 85 | 0.12145 | 61 | 0.971 | 41 | 233-479 | 897 | 92 |
| ndhH-rps15 | 54 | 0.09918 | 48 | 0.964 | 24 | 96-140 | 176 | 92 |
| ndhI-ndhA | 56 | 0.08949 | 49 | 0.965 | 29 | 82-108 | 121 | 92 |
| ndhJ-ndhK | 28 | 0.18346 | 26 | 0.902 | 15 | 88-161 | 239 | 92 |
| petA-psbJ | 117 | 0.16702 | 66 | 0.982 | 55 | 407-1125 | 2311 | 92 |
| petBintron | 312 | 0.09302 | 84 | 0.996 | 171 | 730-815 | 1170 | 92 |
| petB-petD | 103 | 0.12293 | 64 | 0.985 | 56 | 188-214 | 249 | 92 |
| petDintron | 354 | 0.09224 | 86 | 0.998 | 177 | 692-838 | 1266 | 92 |
| petD-rpoA | 90 | 0.14358 | 64 | 0.979 | 40 | 122-279 | 1017 | 92 |
| petG-trnW | 73 | 0.13439 | 49 | 0.947 | 34 | 96-160 | 294 | 92 |
| petL-petG | 153 | 0.14834 | 73 | 0.992 | 75 | 164-197 | 298 | 92 |
| petN-psbM | 127 | 0.12703 | 68 | 0.988 | 60 | 296-1025 | 1641 | 92 |
| psaA-ycf3 | 214 | 0.10356 | 73 | 0.991 | 94 | 527-887 | 1597 | 92 |
| psaB-psaA | 2 | 0.01191 | 3 | 0.279 | 1 | 25 | 25 | 92 |
| psaC-ndhE | 188 | 0.14237 | 78 | 0.995 | 85 | 217-439 | 570 | 92 |
| psaI-ycf4 | 98 | 0.1405 | 65 | 0.966 | 43 | 230-502 | 896 | 92 |
| psaJ-rpl33 | 81 | 0.07696 | 63 | 0.982 | 32 | 255-673 | 1096 | 92 |
| psbA-trnK | 67 | 0.04954 | 61 | 0.974 | 31 | 150-298 | 435 | 92 |
| psbB-psbT | 15 | 0.05348 | 20 | 0.838 | 7 | 97-199 | 345 | 92 |
| psbC-trnS | 105 | 0.18819 | 65 | 0.978 | 52 | 193-304 | 574 | 92 |
| psbE-petL | 222 | 0.09112 | 80 | 0.995 | 95 | 570-1426 | 2508 | 92 |
| psbF-psbE | 9 | 0.13471 | 10 | 0.732 | 3 | 9-15 | 15 | 92 |
| psbH-petB | 75 | 0.10173 | 61 | 0.966 | 40 | 127-201 | 313 | 92 |
| psbI-trnS-GCU | 29 | 0.22813 | 40 | 0.92 | 14 | 55-209 | 338 | 92 |
| psbJ-psbL | 81 | 0.0979 | 58 | 970 | 37 | 134-176 | 248 | 92 |
| psbK-psbI | 33 | 0.20046 | 34 | 0.876 | 14 | 113-432 | 830 | 92 |
| psbL-psbF | 11 | 0.11745 | 12 | 0.746 | 6 | 22 | 24 | 92 |
| psbM-trnD | 112 | 0.10424 | 72 | 0.989 | 60 | 548-1285 | 2256 | 92 |
| psbN-psbH | 5 | 0.05867 | 6 | 0.534 | 3 | 10-121 | 157 | 92 |
| psbT-psbN | 5 | 0.03307 | 7 | 0.167 | 2 | 7-78 | 96 | 92 |
| psbZ-trnG | 105 | 0.12331 | 65 | 0.981 | 47 | 238-969 | 1906 | 92 |
| rbcL-accD | 203 | 0.10764 | 75 | 0.933 | 85 | 502-898 | 3988 | 92 |
| rpl14-rpl16 | 39 | 0.14139 | 46 | 0.936 | 22 | 108-170 | 262 | 92 |
| rpl16intron | 429 | 0.09968 | 89 | 0.999 | 217 | 864-1096 | 1804 | 92 |
| rpl16-rps3 | 60 | 0.13317 | 49 | 0.94 | 29 | 122-206 | 293 | 92 |
| rpl20-rps12 | 365 | 0.10193 | 85 | 0.998 | 177 | 732-862 | 1362 | 92 |
| rpl22-rps19 | 0 | 0 | 1 | 0 | 0 | 37-189 | 474 | 92 |
| rpl23-trnI(CAU) | 76 | 0.05218 | 46 | 0.942 | 39 | 160-239 | 405 | 92 |
| rpl32-trnL(UAG) | 99 | 0.15755 | 63 | 0.973 | 47 | 238-1265 | 1841 | 92 |
| rpl33-rps18 | 31 | 0.2192 | 31 | 0.914 | 12 | 57-440 | 749 | 92 |
| rpoA-rps11 | 40 | 0.06713 | 37 | 0.908 | 18 | 56-136 | 684 | 92 |
| rpoB-trnC | 351 | 0.1429 | 85 | 0.998 | 165 | 930-1523 | 2679 | 92 |
| rpoC1intron | 361 | 0.08433 | 85 | 0.998 | 171 | 686-778 | 1201 | 92 |
| rpoC2-rpoC1 | 105 | 0.1454 | 67 | 0.99 | 51 | 140-618 | 658 | 92 |
| rps2-rpoC2 | 70 | 0.10955 | 56 | 0.973 | 41 | 204-329 | 486 | 92 |
| rps4-trnT | 43 | 0.113492 | 40 | 0.92 | 23 | 118-466 | 847 | 92 |
| rps7-rps12(3') | 9 | 0.01352 | 10 | 0.605 | 2 | 53 | 53 | 92 |
| rps8-rpl14 | 73 | 0.16824 | 64 | 0.968 | 34 | 135-426 | 803 | 92 |
| rps11-rpl36 | 30 | 0.07056 | 32 | 0.904 | 14 | 54-145 | 226 | 92 |
| rps12(3')intron | 75 | 0.01548 | 49 | 0.966 | 36 | 539-608 | 656 | 92 |
| rps12(3')-trnV(GAC) | 131 | 0.02437 | 51 | 0.933 | 65 | 1304-1951 | 2032 | 92 |
| rps12-clpP | 77 | 0.12962 | 58 | 0.98 | 38 | 148-486 | 638 | 92 |
| rps12-clpP | 52 | 0.069 | 43 | 0.925 | 24 |  | 217 | 92 |
| rps15-ycf1 | 5 | 0.44036 | 8 | 0.687 | 3 | 197-2920 | 3469 | 92 |
| rps18-rpl20 | 11 | 0.26705 | 14 | 0.758 | 6 | 172-361 | 592 | 92 |
| rps19-rpl2 | 43 | 0.1279 | 34 | 0.888 | 25 | 54-83 | 124 | 92 |
| rrn4.5-rrn5 | 62 | 0.02695 | 29 | 0.804 | 32 | 214-247 | 478 | 92 |
| rrn5-trnR(ACG) | 85 | 0.05108 | 41 | 0.903 | 38 | 235-276 | 367 | 92 |
| rrn16-trnI(GAU) | 120 | 0.05728 | 42 | 0.897 | 47 | 248-485 | 553 | 92 |
| rrn23-rrn4.5 | 23 | 0.02062 | 22 | 0.807 | 8 | 96-106 | 109 | 92 |
| trnA(UGC)intron | 119 | 0.01802 | 50 | 0.968 | 66 | 690-817 | 898 | 92 |
| trnA(UGC)-rrn23 | 24 | 0.01676 | 25 | 0.866 | 7 | 124-164 | 190 | 92 |
| trnC-petN | 130 | 0.12758 | 71 | 0.983 | 63 | 409-933 | 1600 | 92 |
| trnD(GUC)-trnY(GUA) | 150 | 0.18046 | 79 | 0.992 | 69 | 388-553 | 1031 | 92 |
| trnE-trnT | 112 | 0.1562 | 61 | 0.977 | 51 | 220-1047 | 2203 | 92 |
| trnF-ndhJ | 225 | 0.20374 | 79 | 0.996 | 117 | 416-904 | 1611 | 92 |
| trnfM(CAU)-rps14 | 123 | 0.14905 | 72 | 0.99 | 59 | 151-210 | 277 | 92 |
| trnG(GCC)intron | 465 | 0.10645 | 87 | 0.998 | 212 | 711-850 | 1274 | 92 |
| trnG(GCC)-trnR(UCU) | 12 | 0.17774 | 14 | 0.742 | 3 | 110-429 | 729 | 92 |
| trnG-trnfM | 127 | 0.18375 | 71 | 0.989 | 57 | 150-197 | 366 | 92 |
| trnH(GUG)-psbA | 45 | 0.18292 | 47 | 0.95 | 23 | 127-582 | 1030 | 92 |
| trnI(GAU)intron | 130 | 0.01426 | 49 | 0.959 | 46 | 721-957 | 1071 | 92 |
| trnI(GAU)-trnA(UGC) | 14 | 0.04305 | 12 | 0.671 | 11 | 56-75 | 89 | 92 |
| trnK-matK | 102 | 0.09859 | 69 | 0.977 | 52 | 225-313 | 499 | 92 |
| trnL(CAA)-ndhB | 119 | 0.02521 | 52 | 0.955 | 56 | 550-618 | 791 | 92 |
| trnL(UAA)intron | 189 | 0.08651 | 80 | 0.995 | 85 | 420-553 | 845 | 92 |
| trnL(UAA)-trnF(GAA) | 40 | 0.2043 | 31 | 0.868 | 18 | 151-440 | 701 | 92 |
| trnL(UAG)-ccsA | 113 | 0.2071 | 66 | 0.973 | 49 | 88-139 | 230 | 92 |
| trnM-atpE | 92 | 0.14179 | 66 | 0.984 | 51 | 153-293 | 512 | 92 |
| trnN(GUU)-ycf1 | 53 | 0.02279 | 39 | 0.921 | 25 | 256-329 | 556 | 92 |
| trnP-psaJ | 88 | 0.12709 | 58 | 0.967 | 41 | 179-542 | 1206 | 92 |
| trnQ(UUG)-psbK | 210 | 0.15308 | 80 | 0.994 | 88 | 322-486 | 853 | 92 |
| trnR(ACG)-trnN(GUU) | 8 | 0.06707 | 9 | 0.349 | 4 | 194-641 | 866 | 92 |
| trnR(UCU)-atpA | 82 | 0.19823 | 57 | 0.951 | 34 | 224-584 | 1021 | 92 |
| trnS(GCU)-trnG(GCC) | 149 | 0.20846 | 75 | 0.989 | 65 | 510-884 | 1905 | 92 |
| trnS-rps4 | 103 | 0.16124 | 58 | 0.961 | 49 | 217-362 | 607 | 92 |
| trnT-trnL | 128 | 0.13725 | 67 | 0.985 | 64 | 600-1897 | 3560 | 92 |
| trnV(GAC)-rrn16 | 55 | 0.02985 | 34 | 0.899 | 31 | 218-242 | 279 | 92 |
| trnV(UAC)intron | 352 | 0.08505 | 88 | 0.999 | 172 | 586-626 | 838 | 92 |
| trnV-trnM | 111 | 0.10696 | 64 | 0.983 | 59 | 157-237 | 366 | 92 |
| trnW-trnP | 0 | 0 | 1 | 0 | 0 | 31-223 | 377 | 92 |
| trnY(GUA)-trnE(UUC) | 26 | 0.07888 | 31 | 0.826 | 13 | 43-86 | 91 | 92 |
| ycf3intron1 | 302 | 0.06737 | 82 | 0.996 | 147 | 697-871 | 1258 | 92 |
| ycf3intron2 | 383 | 0.0874 | 84 | 0.996 | 197 | 719-784 | 988 | 92 |
| ycf3-trnS | 102 | 0.09232 | 69 | 0.986 | 50 | 223-919 | 1547 | 92 |
| ycf4-cemA | 0 | 0 | 1 | 0 | 0 | 27-1054 | 1798 | 92 |
