# Supplementary material for: Comparative analysis of chloroplast genome structure and molecular dating in Myrtales
Source: BMC Plant Biol. 2021 May 15;21:219. doi: 10.1186/s12870-021-02985-9 (PMC8122561; doi:10.1186/s12870-021-02985-9)
Supplement: Supplementary file 4 — Additional file 4: Table S2. Species information and chloroplast genomes GenBank accession number of Outgroups in this study. [file 12870_2021_2985_MOESM4_ESM.docx]

**Species information and lastomes GenBank accession number of Outgrous in Figure S6**

| **Order** | **Family** | **Species name** | **lastomes GenBank accession number** |
| --- | --- | --- | --- |
| Geraniales | Francoaceae | *Viviania marifolia* | NC_023259.1 |
| Geraniales | Geraniaceae | *Pelargonium tetragonum* | NC_031205.1 |
| Geraniales | Geraniaceae | *Pelargonium quercifolium* | NC_031203.1 |
| Crossosomatales | Staphyleaceae | *Staphylea trifolia* | MK488092.1 |
| Sapindales | Rutaceae | *Zanthoxylum tragodes* | NC_046747.1 |
| Sapindales | Meliaceae | *Toona ciliata* | NC_039592.1 |
| Sapindales | Simaroubaceae | *Ailanthus altissima* | NC_037696.1 |
| Huerteales | Dipentodontaceae | *Dipentodon sinicus* | MN275517.1 |
| Huerteales | Tapisciaceae | *Tapiscia sinensis* | NC_036960.1 |
| Brassicales | Brassicaceae | *Yosemitea repanda* | MK637830.1 |
| Brassicales | Brassicaceae | *Shehbazia tibetica* | MK637829.1 |
| Brassicales | Brassicaceae | *Pseudofortuynia leucoclada* | MK637828.1 |
| Malvales | Malvaceae | *Thespesia populnea* | NC_048518.1 |
| Malvales | Malvaceae | *Malvastrum coromandelianum* | MK860037.1 |
| Malvales | Malvaceae | *Hibiscus cannabinus* | NC_045873.1 |
| Fabales | Fabaceae | *Bauhinia brachycarpa* | NC_037762.1 |
| Fabales | Fabaceae | *Spatholobus pulcher* | MT396269.1 |
| Fabales | Fabaceae | *Vicia ramuliflora* | MN758738.1 |
| Fabales | Fabaceae | *Abrus pulchellus* | MT328396.1 |
| Fabales | Fabaceae | *Biancaea sappan* | MN933929.1 |
